# Identification of a ubiquitin-binding domain protein, CD2AP, in predicting the prognosis and treatment of lung adenocarcinoma

**DOI:** 10.3389/fimmu.2025.1726531

**Published:** 2025-12-04

**Authors:** Qinjin Dai, Li Li, Mingfeng He, Jin Huang, Zhixiang Bo, Jishan Yin, Ying Ye

**Affiliations:** 1 Ophthalmology Medical Center, The First Affiliated Hospital of Chongqing Medical University, Chongqing Key Laboratory for the Prevention and Treatment of Major Blinding Eye Diseases, Chongqing Branch (Municipality Division) of National Clinical Research Centre for Ocular Diseases, Chongqing, China; 2 Department of Cardiothoracic Surgery, The Second Affiliated Hospital of Chongqing Medical University, Chongqing, China; 3 Department of Oncology, The Second Affiliated Hospital of Chongqing Medical University, Chongqing, China

**Keywords:** lung adenocarcinoma, ubiquitination-related molecules, CD2-associated protein, drug sensitivity, molecular docking

## Abstract

**Background:**

Lung adenocarcinoma remains a leading cause of cancer mortality, necessitating novel prognostic biomarkers and therapeutic targets. Ubiquitination, a crucial post-translational modification, is deeply implicated in tumourigenesis. This study aims to identify key ubiquitination-related regulators in LUAD and investigate their clinical significance, with a particular focus on *CD2AP*.

**Methods:**

We analysed transcriptomic data from TCGA and GEO databases to identify survival-related ubiquitination genes. Proteomic data from the CPTAC database validated key findings. Functional enrichment, immune cell infiltration, and single-cell RNA sequencing (scRNA-seq) analyses were performed to explore the role of *CD2AP*. Drug sensitivity and molecular docking were used to identify potential therapeutics. Experimental validation included qPCR, Western Blot, immunofluorescence, and functional assays in A549 cells.

**Results:**

*CD2AP* was identified as a central regulator, with its mRNA and protein levels significantly elevated in LUAD tissues, and this elevation was associated with poor survival. *CD2AP* expression correlated with TMB, immune infiltration (particularly monocytes/macrophages), and advanced T stage. scRNA-seq confirmed *CD2AP* enrichment in monocytes and revealed enhanced communication between *CD2AP*+ tumour cells and monocytes. Two drugs, afatinib and dasatinib, were identified as potential *CD2AP*-targeting agents via molecular docking. Functional experiments confirmed that silencing *CD2AP* significantly suppressed the proliferation and migration of A549 cells.

**Conclusions:**

Our study identifies *CD2AP* as a key oncoprotein in LUAD. Our findings suggest that targeting *CD2AP* represents a promising therapeutic strategy for patients with LUAD. Integrating *CD2AP* assessment into clinical practice may enhance personalised treatment planning and prognostic evaluation for patients with LUAD.

## Introduction

Lung cancer is the most prevalent malignancy worldwide, with rising incidence and mortality rates. Despite recent advances in molecular oncology, lung cancer prognosis remains poor, marked by a 5-year survival rate below 15% (1). Lung adenocarcinoma (LUAD) is the predominant histological subtype, comprising 50% to 70% of surgically resected lung cancers (2). LUAD is often diagnosed at an advanced stage due to a lack of early symptoms (3). Over the past decade, treatment has evolved from standard chemotherapy to incorporating targeted therapies, improving survival but not substantially altering overall prognosis. Novel therapeutic targets and effective prognostic models are urgently needed to improve LUAD outcomes.

Ubiquitin (Ub) is a small molecular protein that exists in most eukaryotic cells and contains 76 amino acid residues (4). Ubiquitination is the process by which ubiquitin molecules bind to proteins, thereby regulating the stability and function of the substrate protein, and is an important type of PTM (5). Ubiquitin or ubiquitin-like proteins (UBLs) bind to substrate proteins and then target them for proteasomal degradation or nondegradation signalling (6). The ubiquitin on the modified protein is covalently bound to lysine side chain residues through a series of enzymatic reactions involving binding to ubiquitin-activating enzyme E1,ubiquitin-conjugating enzyme E2, and ubiquitin ligase enzyme E3 (7). The system, consisting of ubiquitin-binding domains (UBDs), ubiquitin, UBLs, substrate proteins, ubiquitinating enzymes, and proteasomes, is collectively called the ubiquitination network. The regulation of the ubiquitination network depends on a family of receptor proteins, known as ubiquitin-binding proteins (UBPs), that can specifically recognise ubiquitin chains or ubiquitin monomers of varying lengths and modifications. UBPs often contain one or more UBDs, which can recognise and bind various ubiquitination modifications and transmit signals, thereby determining the specificity of substrate protein functions (8). UBDs are small in molecular weight, mostly between 20 and 150 amino acids, and can fold independently to form stable structures to directly bind mono- or poly-ubiquitinated substrates in the form of noncovalent bonds (9). Therefore, UBDs play a pivotal role in the ubiquitination signalling network.

In this study, a key UBP, CD2-associated protein (CD2AP), which contains UBDs, was identified. Its gene is located on chromosome 6, comprises 18 exons, encodes a 639-amino-acid protein, and has a molecular weight of 80 kDa (10). CD2AP mainly consists of three consecutive Src homology 3 (SH3) domains near the N-terminus, a central proline-rich region, and a complex domain at the C-terminus. These domains include multiple actin- and membrane protein-binding sites, enabling regulation of the cytoskeleton through interactions with actin and membrane proteins, and mediating receptor endocytosis and vesicle trafficking (11). CD2AP is found in various tissues, with the highest expression in renal podocytes. Previous studies have demonstrated that CD2AP interacts with other proteins and plays a crucial role in maintaining glomerular filtration (12). Moreover, mutations in the *CD2AP* gene are associated with the development of Alzheimer’s Disease (AD) (13). However, the functions of *CD2AP* in cancers remain largely unknown.

Protein ubiquitination and deubiquitination have been shown to play a crucial role in the comprehensive progression of tumours. Dysregulation of ubiquitination can alter the regulation of intracellular physiological processes, potentially leading to the development of cancer. Recent studies have shown that various E2, E3, and deubiquitinating enzymes (DUBs) modulate tumour-promoting gene expression and play significant roles in tumour invasion and metastasis (14–16). Despite the critical role of ubiquitination in cellular regulation, its systematic exploration in LUAD, particularly as a source of prognostic biomarkers and therapeutic targets, remains incomplete. More specifically, the function of ubiquitin-binding domain (UBD) proteins, which act as key decoders of the ubiquitin code, is vastly understudied in the context of LUAD progression and its immunosuppressive microenvironment. This gap is particularly relevant given that ubiquitination modulates key immune pathways, and its dysregulation may contribute to the limited efficacy of current immunotherapies in a subset of patients. To address this, we hypothesised that key UBD-containing proteins drive LUAD pathogenesis by influencing both tumour cell-intrinsic properties and the tumour immune microenvironment, and that such proteins could serve as novel prognostic biomarkers and therapeutic targets.

To test this hypothesis, we conducted a comprehensive study to elucidate the role of ubiquitination-related regulators in lung adenocarcinoma. To this end, we first employed a machine learning approach to construct a robust ubiquitination-related prognostic signature from a candidate gene pool. Through a stepwise screening and validation pipeline, we identified the ubiquitin-binding domain-containing protein CD2AP as a central regulator within this signature. We then subjected *CD2AP* to rigorous multi-omics and experimental validation to delineate its oncogenic functions, impact on the tumour immune microenvironment, and therapeutic potential. This strategy, transitioning from a multigene model to a core molecular determinant, ensures a comprehensive and unbiased discovery of key drivers in lung adenocarcinoma pathogenesis.

## Materials and methods

### Data collection

We acquired the transcriptome sequencing data of 497 LUAD samples, 54 normal lung tissues, and the clinical features of 454 LUAD patients from The Cancer Genome Atlas (TCGA) database (https://portal.gdc.cancer.gov/). The RNA-seq data and the clinical information of 442 LUAD patients in the validation cohort were downloaded from the Gene Expression Omnibus (GEO) database (https://www.ncbi.nlm.nih.gov/geo/, GSE72094). All of the RNA-seq data were downloaded in the form of fragments per kilobase of transcript per million mapped reads (FPKM). Before cross-validation, the gene expression data were normalised using the “Scale” function to minimise deviations caused by the diversity of sample batches and sequencing platforms. We extracted these ubiquitination regulators from the iUUCD (integrated annotations for Ubiquitin and Ubiquitin-like Conjugation Database, version 2.0; URL: http://iuucd.biocuckoo.org/) database, and 807 ubiquitination-related genes in Homo sapiens were screened out (**Supplementary Table S1**).

The proteomics data were obtained from The ProteoCancer Analysis Suite (PCAS) platform (https://jingle.shinyapps.io/PCAS/) (17).

For experimental validation, this study utilised a cohort of 13 LUAD patients, from whom both tumour tissues and matched adjacent normal tissues were collected. The samples were allocated as follows: tumour and paired normal tissues from 5 patients were subjected to quantitative real-time PCR (RT-qPCR), samples from another five patients were used for Western Blot analysis, and tissues from the remaining three patients were employed for immunofluorescence assays. The diagnosis of LUAD for all included patients was pathologically confirmed. The research protocol received approval from the Ethics Committee of the Second Affiliated Hospital of Chongqing Medical University (Approval Number: 2025-282). It was conducted in accordance with the principles outlined in the Declaration of Helsinki. The clinicopathological characteristics of the 13 patients were shown in **Supplementary Table S2**.

### Screening out DEGs and SRGs

We constructed a volcano plot using the “GEOquery”, “limma”, “ggplot2”, “ggrepel”, and “ggthemes” R packages to display the expression levels of ubiquitination-related genes. The “limma” R package was applied to identify the DEGs between normal and tumour tissues, with the criteria of false discovery rate (FDR)<0.05 and |log2 FC|>1 as the previous studies described (18).

Combined with the full clinical information of 454 LUAD patients in the TCGA dataset, survival-related genes (SRGs) were identified using the univariate Cox regression model with *P* values < 0.05 (“survival” R package). To screen the intersecting genes between DEGs and SRGs, a Venn diagram was constructed by applying the “VennDiagram” R package.

To explore the connections among the 12 intersecting genes, Spearman correlation analysis was conducted by employing the “reshape2” R package, and a PPI network was constructed by using the Search Tool for the Retrieval of Interacting Genes (STRING), version 11.0 (URL: https://www.string-db.org/).

### The machine-learning method

We applied the least absolute shrinkage and selection operator (LASSO) Cox regression model included in the “glmnet” R package to determine the key regulators. Finally, after 1,000 computational simulations, 5 genes with nonzero coefficients were retained based on the minimum criteria. The risk score was calculated by the formula: Risk score= $\sum_{i}^{5}X_{i}\times Y_{i}$ (X: gene expression level; Y: gene’s coefficient). Before calculating the risk score, the expression data of each gene was normalised by the “Scale” function in both TCGA and GEO cohorts. According to the median score of the training cohort, the patients in both the training and validation cohorts were divided into low- or high-risk groups. We applied the “ggpolt2” and “Rtsne” R packages to perform principal component analysis (PCA) and t-distributed stochastic neighbour embedding (t-SNE) analysis, illustrating the discrepancies in gene expression between the low- and high-risk groups. A time-dependent ROC curve (1-, 3-, and 5-year) was constructed using the “survival”, “survminer”, and “timeROC” R packages to evaluate the sensitivity and specificity of the risk score.

### Mutation and immune cell analysis

The simple nucleotide variation (SNV) data were obtained from the TCGA database, and the “Maftools” package in R was used to perform the gene mutation analysis. The infiltration fraction of each immune cell was calculated by the “CIBERSORT” R package, and the somatic copy number alteration (sCNA) analysis was performed by the TIMER online tool (version 2.0, http://timer.comp-genomics.org/) (19).

### Functional enrichment analysis

We explored the functions of DEGs between the *CD2AP*-high and *CD2AP*-low groups by using Gene Ontology (GO) and Kyoto Encyclopedia of Genes and Genomes (KEGG) analyses performed by the “cluster Profiler” R package. Those DEGs were screened out according to the criteria of |log_2_ FC| ≥ 1 and FDR < 0.05. The Gene Set Enrichment Analysis (GSEA) was also conducted by the “cluster Profiler” R package.

To quantify pathway activity in individual samples, we performed single-sample gene set enrichment analysis (ssGSEA). This was accomplished using the GSVA R package with its built-in “ssGSEA” method. The resulting ssGSEA scores represent the enrichment level of each pathway in each sample. Finally, we investigated the correlation between *CD2AP* expression and pathway scores by Spearman correlation analysis.

### Single-cell analysis

The corresponding single-cell data files (.h5 format) and annotation results were downloaded from the Tumour Immune Single-cell Hub 2 (TISCH2, http://tisch.comp-genomics.org/, GSE99254). The single-cell data were then processed and analysed using the R software MAESTRO and Seurat, and cell clustering and sub-clustering were performed using the t-SNE method.

For our subsequent analysis, we employed the single-cell RNA sequencing data from Bischoff et al., which includes samples from 10 normal tissues and 10 LUAD tissues (20); the original data are accessible here: https://codeocean.com/capsule/8321305/tree/v1. We performed dimensionality reduction and analysis using the Seurat package in R. Quality control was implemented by applying a gene count threshold of 200 to 4000 and excluding cells with a mitochondrial gene content of less than 10%. Following data normalisation, principal component analysis (PCA) was used to capture primary sources of variation, and batch effects were corrected using the “limma” and “sva” packages. Cells were clustered using the Louvain algorithm, and the resulting clusters were visualised in two dimensions using Uniform Manifold Approximation and Projection (UMAP). We then conducted a refined sub-clustering analysis on these populations based on canonical monocyte markers. Finally, to decipher intercellular communication, we utilised the CellChat package to identify significant ligand-receptor interactions, thereby predicting potential ligand-receptor pairs and evaluating the strength and directionality of these interactions across distinct cell clusters to reveal key signalling pathways and communication trends.

### Drug-sensitive analysis and molecular docking

To explore the potential sensitive drugs in dealing with *CD2AP* enrichment, we applied the Gene Set Cancer Analysis (GSCA, https://guolab.wchscu.cn/GSCA/#/) database, which includes both the GDSC (Genomics of Drug Sensitivity in Cancer) and CTRP (Cancer Therapeutics Response Portal) database (21).

To evaluate the binding affinities and modes of interaction between the candidate drugs and CD2AP protein, a silico protein-ligand docking software named CB-Dock2 was utilised (22). The molecular structure of CD2AP was obtained from the PDB (http://www.rcsb.org/pdb/home/home.do) database, while the 3D coordinates and details of the drugs were acquired from the PubChem (https://pubchem.ncbi.nlm.nih.gov/). To accomplish the docking procedures, water molecules were removed from all proteins and molecules, and polar hydrogen atoms were added to the remaining atoms.

### Experimental verification

RT-qPCR: Total RNA was extracted from human tissues using an RNA extraction kit (Accurate Biotechnology, Changsha, China). Subsequently, cDNA was synthesised using the Evo M-MLV Reverse Transcription Kit with gDNA eraser function (Accurate Biotechnology, Changsha, China). Finally, qPCR amplification of the target genes was performed on the QuantStudio™ 5 system (Thermo Fisher Scientific) using SYBR Green qPCR Master Mix (Medcom). The primer sequences used for RT-PCR were as follows: *CD2AP* forward primer (F): 5’-GGCATGGGAATGTAGCAAGTC-3’, *CD2AP* reverse primer (R): 5’-CCACCAGCCTTCTTCTACCTC-3’, β-actin forward primer: 5’-CCTGGCACCCAGCACAAT-3’, β-actin reverse primer: 5’-GGGCCGGACTCGTCATAC-3’. Gene expression levels were quantified using the double internal reference method (2-ΔΔCT method), normalised to the β-actin gene as a control.

Western Blotting (WB): Total protein was extracted from human tissues using RIPA lysis buffer (WSHTBio, Shanghai, China). Proteins were separated on a Future PAGE 4-20% gel (ACE Biotechnology, Hunan, China) and subsequently transferred to a PVDF membrane (Merck Millipore, Burlington, MA, USA). The membrane was blocked at room temperature for 30 minutes with NcmBlot blocking buffer (NCM Biotechnology, Suzhou, China), followed by an overnight incubation at 4 °C with primary antibodies against CD2AP and β-actin. This was followed by a 1-hour incubation with corresponding secondary antibodies at room temperature. After the CD2AP antibody was stripped using a Rapid Antibody Stripping Buffer (Biotides, Beijing, China), the membrane was re-probed for β-actin. Protein bands were visualised using a Bio-Rad gel imaging system and quantitatively analysed using Image Lab software.

Immunofluorescence staining: Immunofluorescence staining for CD2AP was performed on formalin-fixed, paraffin-embedded (FFPE) tissue sections from the lungs. Briefly, sections were cut to a thickness of 4-5 μm, deparaffinised in xylene, and rehydrated through a graded ethanol series. Subsequently, antigen retrieval was performed by heating the sections in 10 mM sodium citrate buffer (pH 6.0) at 95-100 °C for 20 minutes. After cooling and washing with phosphate-buffered saline (PBS), the sections were permeabilised and blocked with PBS containing 0.3% Triton X-100 and 5% normal goat serum for 1 hour at room temperature. The sections were then incubated overnight at 4 °C with a primary antibody against CD2AP (Proteintech, Wuhan, China). This was followed by incubation with an appropriate secondary antibody for 1 hour at room temperature. The sections were mounted with a DAPI-containing mounting medium and imaged using a confocal microscope.

Cell Culture and Transfection: The A549 cell line and its corresponding culture medium were purchased from Procell Life Science & Technology Co., Ltd. Cells were cultured under standard conditions (37 °C, 5% CO_2_, 95% humidity).*CD2AP* Human Pre-designed siRNA was acquired from MedChemExpress (MCE). Transfection was performed using Lipofectamine 3000 reagent according to the manufacturer’s instructions.

Cell Migration Assay: Transwell migration assays and scratch wound-healing migration assays were performed using A549 cells to validate their migration ability. A Transwell migration assay was performed in 24-well Transwell chambers (Costar-Corning, New York, USA) with an 8.0-μm pore polycarbonate filter. The lower chamber was filled with 600 μL of Ham’s F-12K with 10% FBS. A549 cell suspensions (5*10^4^ cells/well) were added to the upper compartment with Ham’s F-12K. After incubating for 24 h, the filters were washed, fixed, and stained with 0.1% crystal violet. The number of cells per field that migrated to the lower surface of the filters was determined microscopically.

For the wound healing migration experiment, A549 cells, in their logarithmic growth phase, were plated in 6-well plates (Corning Incorporated, Corning, NY, USA). Once the cells reached approximately 90% confluence, a linear wound was generated in the cell monolayer by scraping with a sterile 200 μL pipette tip. The cell layer was rinsed three times with PBS to remove dislodged cells and was subsequently maintained in Ham’s F-12K medium supplemented with 2% fetal bovine serum (FBS). Photographs were taken at the 0-hour and 24-hour time points to evaluate the wound closure capability.

CCK8 Assay: Cell viability was assessed using the CCK-8 assay (Xiamen Immocell Biotechnology Co., Ltd., Fujian, China). A549 cells during their logarithmic growth phase were plated in 96-well plates (1*10^4^ cells/well). At 24, 48, and 72 hours, the CCK-8 reagent was added, and after incubation at 37 °C for 2 hours, the absorbance at 450 nm was measured using a microplate reader.

### Statistical analysis

To compare the gene expression between the two groups, we applied Student’s t-test. The Pearson chi-square test was used to recognise differences in the categorical variables. The Kaplan-Meier curve was applied to compare OS time and survival rates between subgroups. Univariate and multivariable Cox regression models were employed to evaluate the independent prognostic value of the risk model. All statistical analyses were accomplished with R software (version 4.1.1).

## Results

### Identification of ubiquitination-related key genes

**Figure 1:**
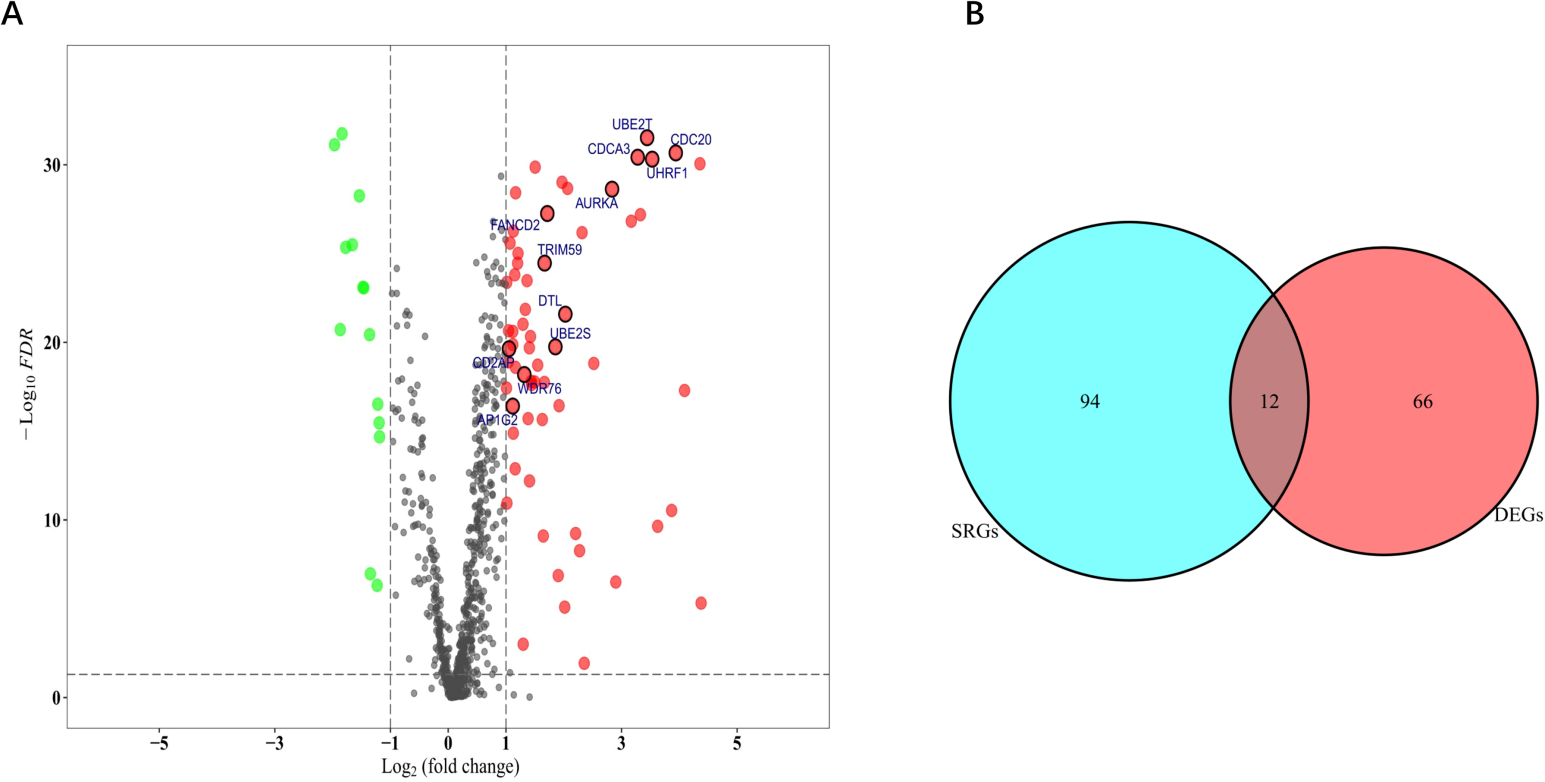
Identification of ubiquitination-related DEGs and SRGs. **(A)** Volcano plot showing the DEGs between normal and tumour tissues (green dot: downregulated at least 2-fold in tumour tissues; red dot: upregulated at least 2-fold in tumour tissues). **(B)** A Venn plot to screen out the intersection genes.

The expression of these ubiquitination-related genes was compared between 54 normal lung tissues and 497 LUAD samples in the TCGA database (only 758 genes were detected), and 582 DEGs were identified (*FDR* < 0.05). The volcano plot of the DEGs is shown in **Figure 1A** (the expression levels of all 758 genes are presented in **Supplementary Table S3**). To filter more strictly, the criteria of |log_2_ FC|C1 were added, and 78 DEGs were screened out.

Combined with the survival information of 454 LUAD patients in the TCGA cohort, we evaluated the prognostic value of the 758 ubiquitination-related genes (**Supplementary Table S4**) and identified 106 genes (survival-related genes, SRGs) that were significantly associated with survival status (*P* < 0.05). The Venn diagram revealed that 12 of them were also DEGs with |log_2_ FC|C1 (**Figure 1B**), and the names of these genes are also emphasised in **Figure 1A** (*DTL*, *UBE2S*, *AURKA*, *TRIM59*, *WDR76*, *UHRF1*, *CD2AP*, *CDCA3*, *CDC20*, *UBE2T*, *FANCD2*, *AP1G2*).

**Figure 2:**
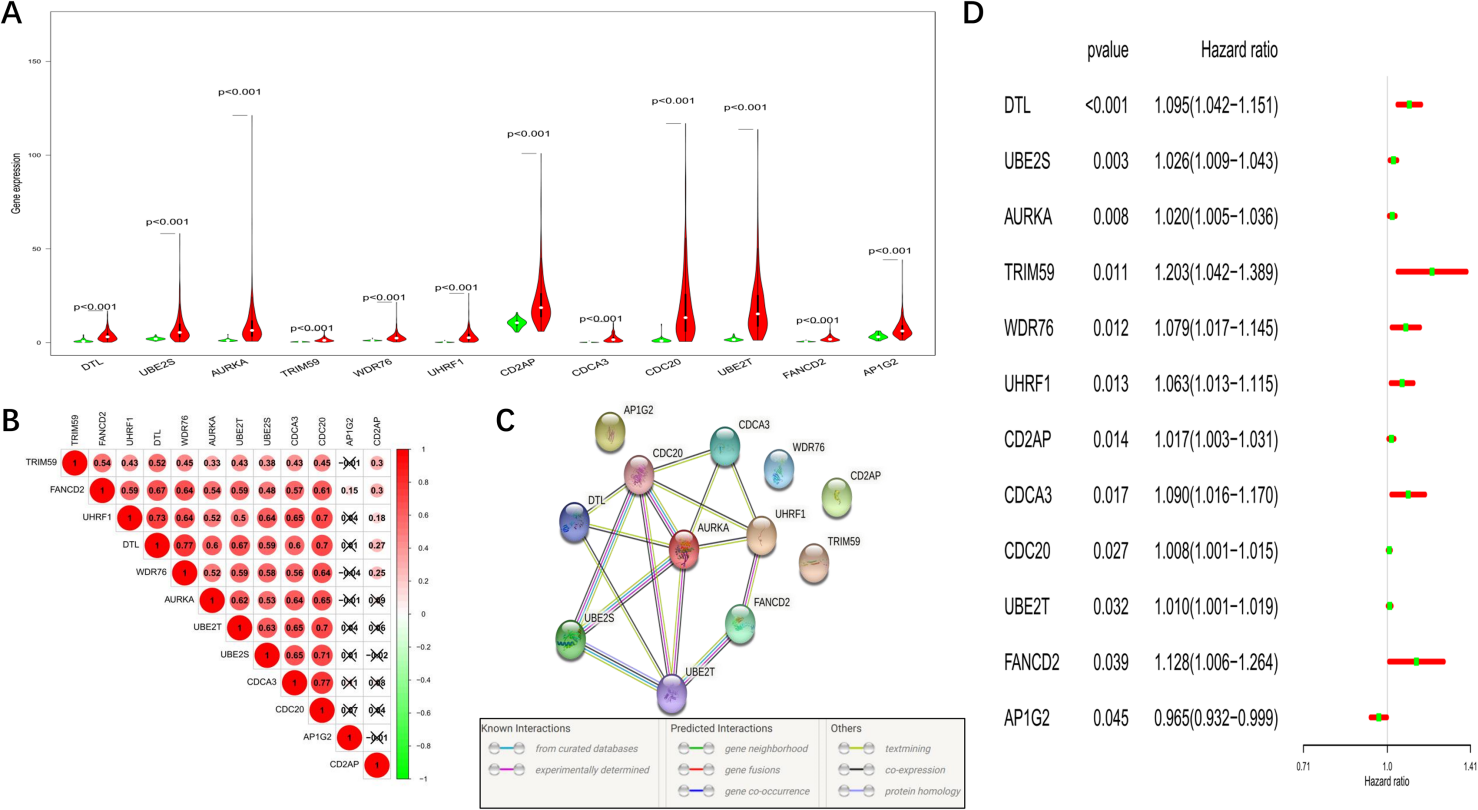
Characteristics of the 12 candidate genes. **(A)** Violin plot comparing the expression levels of the 12 genes between normal and tumour tissues (red: LUAD tissues; green: normal lung tissues). **(B)** Spearman correlation analysis for the 12 genes (red: positive correlation, green: negative correlation). **(C)** PPI network showing the interactions among the 12 genes. **(D)** Forest plot displaying the prognostic value of each candidate gene.

We detected the expression levels of the 12 hub genes and found that they were all upregulated in the tumour tissues (**Figure 2A**). To better understand the correlations among the 12 genes, a Spearman correlation analysis was performed based on the co-expression levels of each gene in the TCGA cohort (red: positive correlation, green: negative correlation, **Figure 2B**). A protein-protein interaction (PPI) network was also constructed to illustrate the relationships among these genes (**Figure 2C**, with a minimum required interaction score of 0.4). In addition, 454 samples with complete survival information were used to establish a forest plot illustrating the prognostic values of these 12 genes (**Figure 2D**).

### Development and validation of a prognostic gene signature by applying the machine learning method

**Figure 3:**
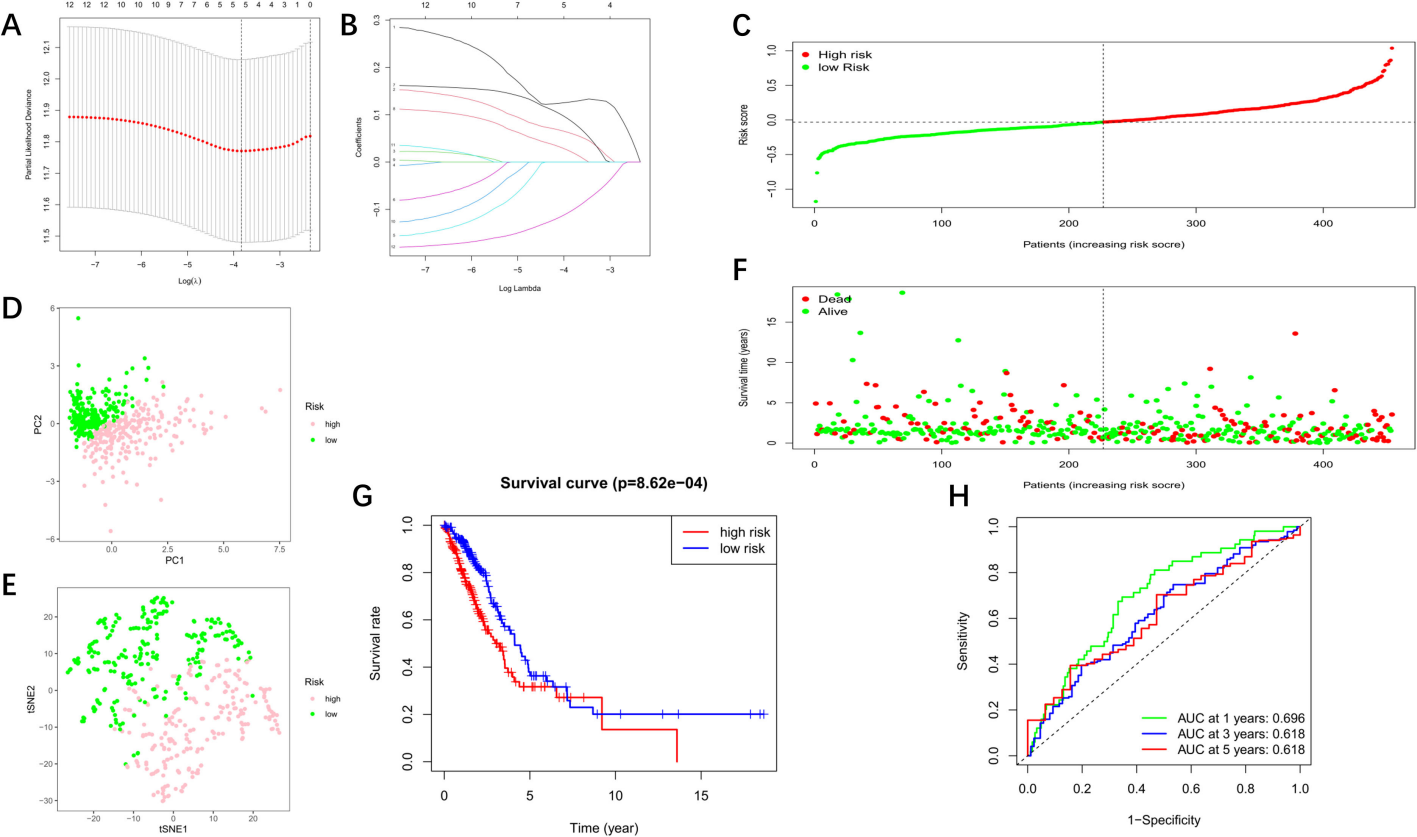
Development of a risk signature based on the TCGA cohort. **(A)** Cross-validation for tuning the parameter selection. **(B)** LASSO regression for the 12 candidate genes. **(C)** The distribution of risk scores for the patients in the training cohort. **(D)** PCA plot for all LUAD patients, categorised by risk groups. **(E)** t-SNE analysis for the two risk groups. **(F)** Survival status for each individual (low-risk: left of the dotted line; high-risk: right of the dotted line). **(G)** Kaplan–Meier curves to compare the OS time between the low- and high-risk groups. **(H)** Time-dependent ROC curves.

By the LASSO machine learning method, only 5 (*AP1G2*, *CD2AP*, *DTL*, *UBE2S*, *CDCA3*) of the 12 candidate genes were retained according to the optimum λ value (**Figures 3A, B**). The risk score could be calculated by the following formula: Risk score = (-0.102*AP1G2 exp) + (0.086*CD2AP exp) + (0.127*DTL exp) + (0.060*UBE2S exp) + (0.021*CDCA3 exp). Each of the 454 LUAD patients then obtained the corresponding risk scores by the formula, and according to the median risk score (-0.0318), all the patients were equally divided into low- and high-risk subgroups (**Figure 3C**). The PCA based on gene expression levels revealed that low- and high-risk patients could be distinctly separated into two clusters (**Figure 3D**), a conclusion verified by t-SNE analysis (**Figure 3E**). A risk plot was constructed, and we found that the number of deaths was significantly higher and the survival time was significantly shorter in the high-risk subgroup (to the right of the dotted line) than in the low-risk group (**Figure 3F**). The Kaplan-Meier curve revealed a significant difference in survival rates between the low- and high-risk groups (*P* < 0.001, **Figure 3G**). Time-dependent receiver operating characteristic (ROC) analysis was used to evaluate the sensitivity and specificity of the risk score. We observed that the area under the curve (AUC) was 0.696 for 1 year, 0.618 for 3 years, and 0.618 for 5 years (**Figure 3H**).

We then applied another GEO dataset (GSE72094) to validate our findings, and the consistent results revealed the robustness of our model (**Supplementary Figure S1**).

### Identification of CD2AP as a key regulator in proteomics analysis

We then applied the CPTAC database to validate our findings at the protein level. To identify the most pivotal candidate from our five-gene signature for further mechanistic and therapeutic exploration, we established the following criteria (1): consistent upregulation at the protein level in LUAD tissues (2), a significant association with poor patient survival in proteomic data, and (3) potential biological novelty and clinical relevance.

**Figure 4:**
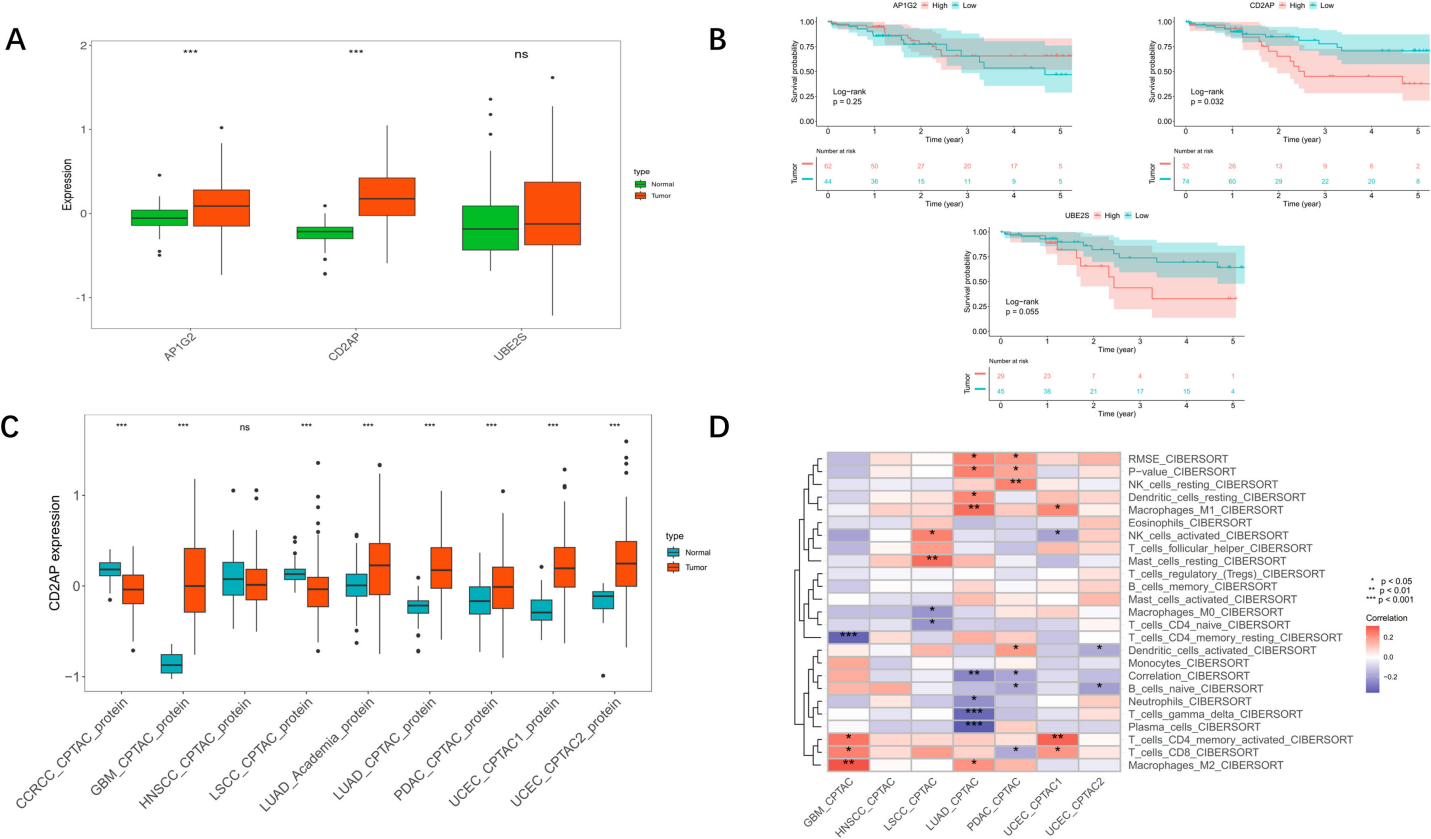
Analysis of proteomics data in the CPTAC database. **(A)** Comparison of the protein levels between normal (green) and tumour (red) samples. **(B)** Kaplan–Meier curve for each protein. **(C)** Pan-cancer analysis for CD2AP (green: normal tissue, red: cancer tissue). **(D)** Relationship between CD2AP protein level and immune infiltration in pan-cancer.

Among the five genes, proteins for CDCA3 and DTL were not retrieved in the CPTAC database. For the remaining three, both AP1G2 and CD2AP proteins were significantly elevated in LUAD samples (**Figure 4A**), fulfilling our first criterion. However, survival analysis based on protein abundance revealed that only CD2AP was significantly associated with poorer overall survival (**Figure 4B**), thereby uniquely satisfying the second and most critical criterion. Furthermore, CD2AP, as a ubiquitin-binding domain (UBD) containing protein, represents a novel and less explored potential regulator in LUAD compared to other cell-cycle related genes in the signature. Its established roles in endocytosis and cytoskeletal remodelling in other diseases provided a compelling biological rationale for its investigation in cancer cell invasion and communication with the tumour microenvironment.

Based on this comprehensive evaluation–protein-level overexpression, prognostic significance, and biological novelty–we identified CD2AP as the central molecule warranting in-depth functional characterisation.

Pan-cancer analysis of CD2AP revealed that its protein levels were evaluated in most types of cancers (**Figure 4C**), except for clear cell renal cell carcinoma (CCRCC), head and neck squamous cell carcinomas (HNSCC), and lung squamous cell carcinoma (LSCC). Moreover, by utilising the CIBERSORT algorithm, we evaluated the correlation between CD2AP protein level and the abundance of immune cells, and the results showed that CD2AP was positively related to M1 macrophages, while being negatively correlated with γδ T-cells and plasma cells in LUAD (**Figure 4D**).

### Exploration of *CD2AP* gene functions in LUAD

As *CD2AP* was identified as a hub gene in our study, we first conducted a pan-cancer analysis to compare the mRNA levels between cancers and the corresponding normal tissues (**Supplementary Figure S2A**). It’s worth noting that in NSCLCs, *CD2AP* had elevated specificity in LUAD. To avoid bias from single-database analysis, we employed the online tool Lung Cancer Explorer (LCE) to conduct a meta-analysis in combination with multiple LUAD databases (23). Seven studies containing 827 LUAD samples and 246 normal tissues were enrolled, although with significant heterogeneity; *CD2AP* was identified as being enriched in tumour samples in most studies (**Supplementary Figure S2B**). Moreover, 2912 LUAD patients from 21 studies with complete survival information were analysed, and the meta-analysis revealed that *CD2AP* was significantly associated with a poor prognosis (**Supplementary Figure S2C**). Next, we constructed the CD2AP-centric PPI network as shown in **Supplementary Figure S2D**. We further explored the correlations between *CD2AP* mRNA levels and clinical features (age, gender, tumour stage, and TNM stages), and the results showed that, compared to the T1 stage, *CD2AP* levels were significantly higher in the advanced stages (T2-T4) (**Supplementary Figure S3**).

**Figure 5:**
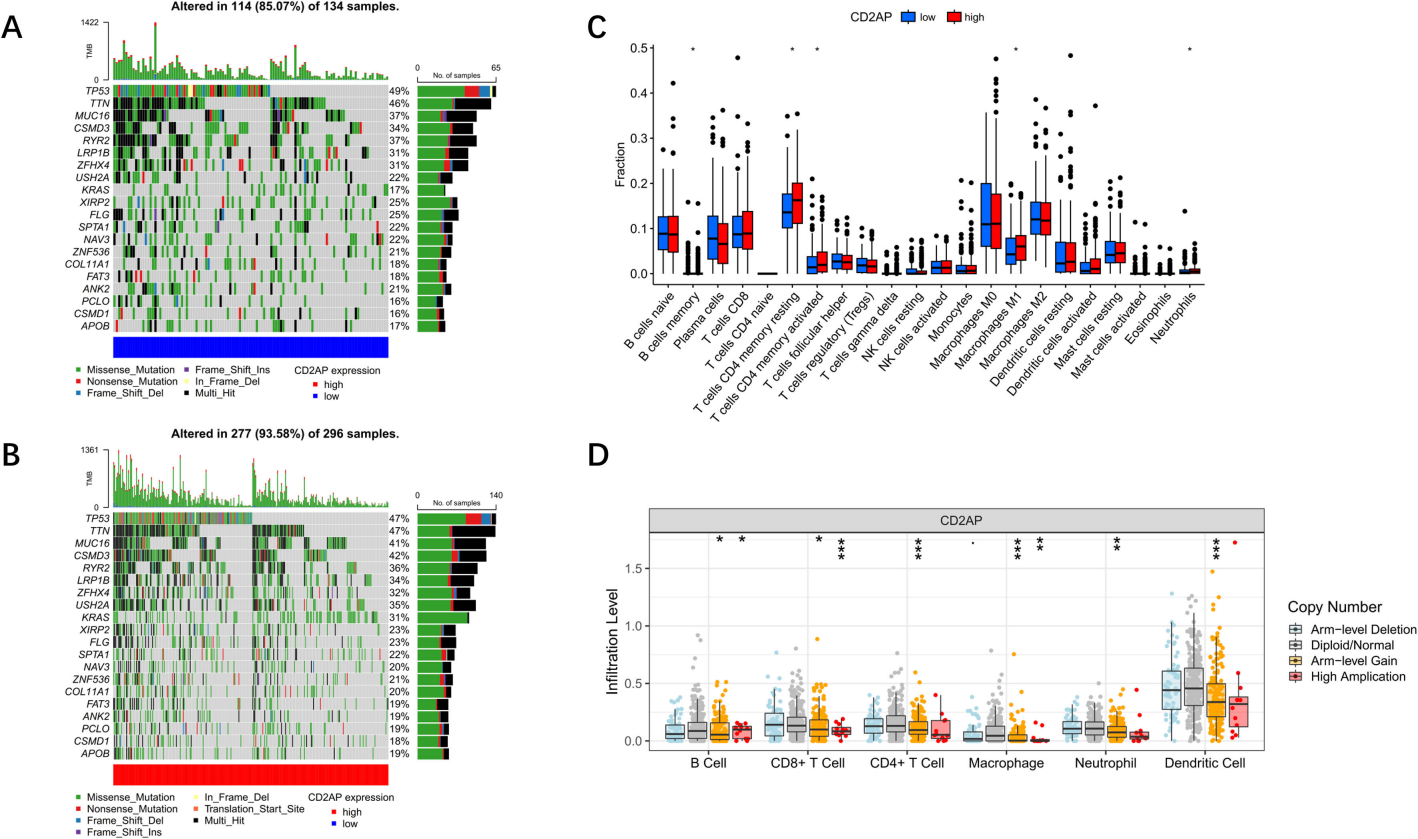
TMB and immune cell analysis. **(A)** The top 20 mutated genes in the *CD2AP*-low subgroup. **(B)** The top 20 mutated genes in the *CD2AP*-high subgroup. In both panels, the genes are ordered from top to bottom by their mutation frequency. The most frequently mutated genes in the entire cohort are TP53 and TTN. **(C)** The infiltration fraction for each immune cell between the *CD2AP*-low(blue) and the *CD2AP*-high(red) subgroups. **(D)** The relationship between *CD2AP* copy number variation and the infiltration level of immune cells. *, *P*<0.05**, *P*<0.01; ***, *P*<0.001.

In the TCGA-LUAD cohort, patients were divided into *CD2AP*-high (N = 296) and *CD2AP*-low (N = 134) subgroups, and the optimal cut-off value was determined based on the lowest log-rank *P*-value in survival analysis. The top 20 commonly mutated genes in LUAD were compared between the *CD2AP*-high and *CD2AP*-low groups, and the results indicated that the gene mutation rate in the *CD2AP*-high group was significantly higher than that in the *CD2AP*-low group (93.58% vs. 85.07%, *P* = 0.004, **Figures 5A, B**). We compared the number of 22 types of immune cells between the *CD2AP*-high and *CD2AP*-low groups and found that the CD4+ T cells and the M1 macrophages were significantly enriched in the *CD2AP*-high subgroup (**Figure 5C**). Moreover, the changes in the copy number of *CD2AP* appeared to significantly affect the immune-infiltration level, especially in macrophages (**Figure 5D**).

### Gene enrichment and pathway analysis

**Figure 6:**
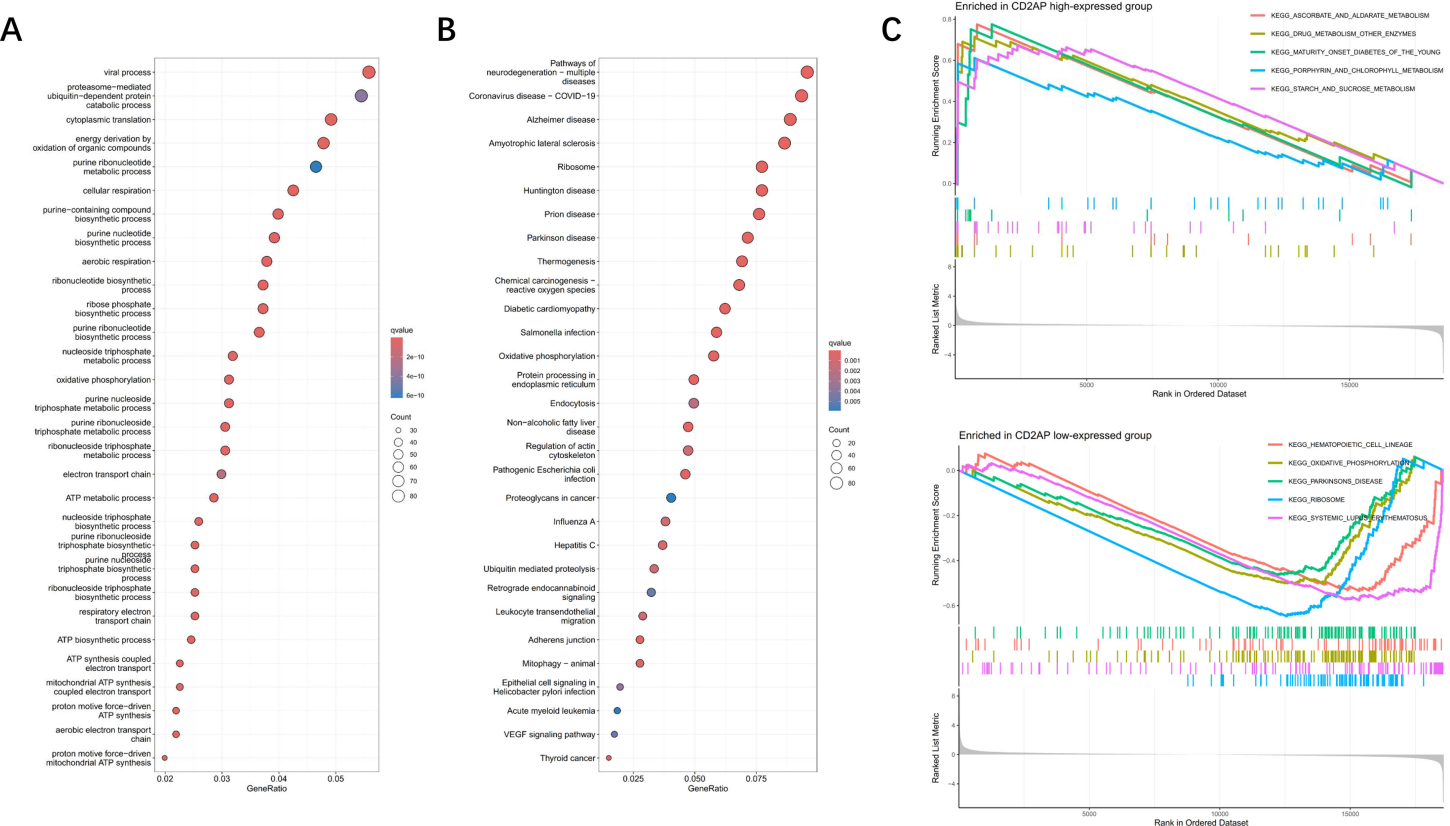
Pathway enrichment analysis. **(A)** GO analysis for the DEGs between the *CD2AP*-low and *CD2AP*-high subgroups. **(B)** KEGG analysis for the DEGs between the *CD2AP*-low and *CD2AP*-high subgroups. **(C)** GSEA analysis for *CD2AP*-low and *CD2AP*-high subgroups.

To screen out the DEGs between the *CD2AP*-high and *CD2AP*-low groups in the TCGA cohort, we utilised the “limma” R package by applying the criteria *FDR* < 0.05 and |log_2_FC | ≥ 2. To explore the functions of these DEGs attributed to the alteration of *CD2AP*, the GO (bubble plot) and KEGG and pathway (bubble plot) analyses were performed. The top 3 gene-enrichment pathways in the GO analysis were the viral process, proteasome-mediated ubiquitin process and cytoplasmic translation (**Figure 6A**). In the KEGG analysis, the DEGs were mainly associated with viral and neurodegenerative diseases, and these disorders were closely related to protein metabolism (**Figure 6B**). We also conducted a GSEA analysis, which showed that the top 5 pathways were associated with metabolism in the *CD2AP* high-expressed group. In contrast, the pathways in the *CD2AP* low-expressed group were mainly related to hematopoietic cells, oxidative phosphorylation, Parkinson’s disease, the ribosome, and systemic lupus erythematosus (**Figure 6C**).

We collected the genes contained in the corresponding pathways and then made a comprehensive correlation analysis between *CD2AP* mRNA levels and 106 common pathways. By utilising the criteria *P*-value < 0.01 and |correlation coefficient| ≥ 0.1, the top 8 pathways were screened out and ranked by correlation coefficient (**Supplementary Figure S4**). The results indicated that *CD2AP* was primarily associated with oxidative phosphorylation, multiple amino acid metabolism, the G2M checkpoint, and the TGF-β pathway.

### Single-cell analysis and cell sub-localisation of CD2AP

**Figure 7:**
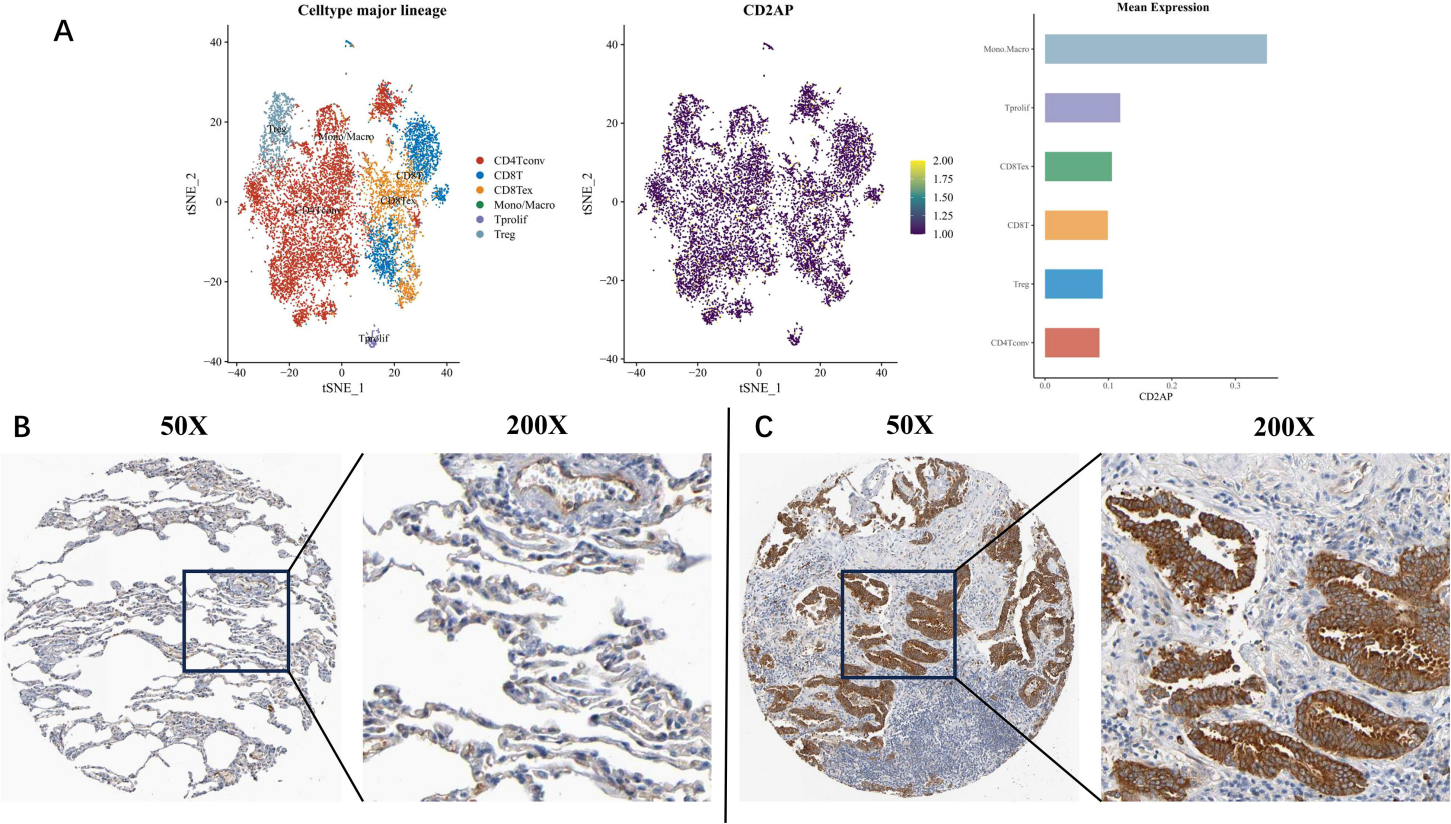
Single-cell and IHC analysis. **(A)** Single-cell analysis for GSE99254. **(B)** Healthy lung tissue (Patient ID: 2101) staining with CD2AP. **(C)** LUAD tissue (Patient ID: 2438) staining with CD2AP.

Our bulk tissue analyses indicated a positive correlation between *CD2AP* expression and macrophage infiltration. To investigate the cellular origin of this correlation, we performed single-cell data analyses. The results revealed that *CD2AP* was not only expressed in tumour cells but was also notably enriched in mononuclear macrophages (**Figure 7A**).

We utilised the Human Protein Atlas (HPA) online tool to determine the subcellular localisation of CD2AP in cells. The healthy lung tissue (Patient ID: 2101) was exhibited in **Figure 7B**, and the staining intensity of CD2AP was identified as weak. Tumour tissue from a LUAD patient (Patient ID: 2438) exhibited a vigorous staining intensity of CD2AP (**Figure 7C**), confirming that the CD2AP protein was predominantly cytoplasmic and membrane-expressed.

**Figure 8:**
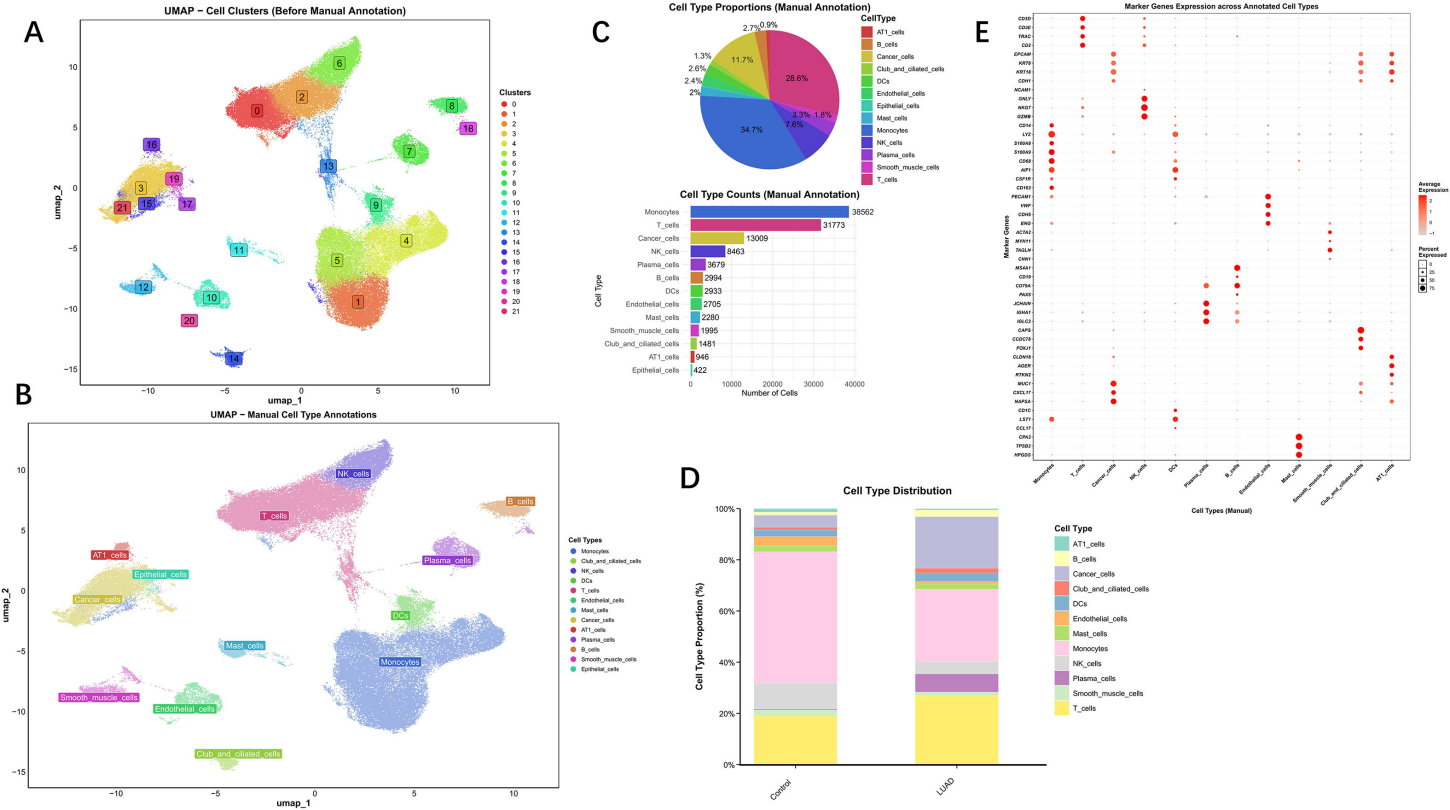
Perform subset analysis on LUAD. **(A)** UMAP plot revealing the 21 clusters of cells in LUAD; **(B)** Marker gene-based cell annotation on UMAP plot; **(C)** Cell type proportions; **(D)** Cell type distributions; **(E)** Marker gene expression profiles of cells.

We next performed a detailed analysis of the single-cell RNA sequencing data from Bischoff et al., which comprised 10 LUAD and 10 normal lung samples. Following initial quality control to remove low-quality cells and genes (**Supplementary Figures S5A, B**), the data were processed. Principal component analysis (PCA) was employed to extract core features, which are visualised in **Supplementary Figure S5C**. Batch effects were mitigated using the Harmony algorithm, resulting in a significant reduction of technical variations between experimental batches. Post-correction, samples from different batches exhibited improved integration in the data space, resulting in the adjusted UMAP visualisation shown in **Supplementary Figure S5D**. Dimensionality reduction and clustering via UMAP identified 21 distinct clusters. Based on canonical marker gene sets, these clusters were annotated into 13 independent lineages: T cells, Epithelial cells, NK cells, Monocytes, Endothelial cells, Smooth muscle cells, B cells, Plasma cells, Club and ciliated cells, AT1 cells, Cancer cells, DCs, and Mast cells (**Figures 8A, B**). Statistical analysis of these annotated cell subpopulations revealed that Monocytes were the most abundant in both cell number and proportion (**Figure 8C**). This predominance of Monocytes was consistently observed within both the normal and LUAD groups (**Figure 8D**). Consequently, subsequent analyses focussed explicitly on the monocyte population. Marker genes for all cell subpopulations are displayed in **Figure 8E**, with a comprehensive list provided in **Supplementary Table S5**.

**Figure 9:**
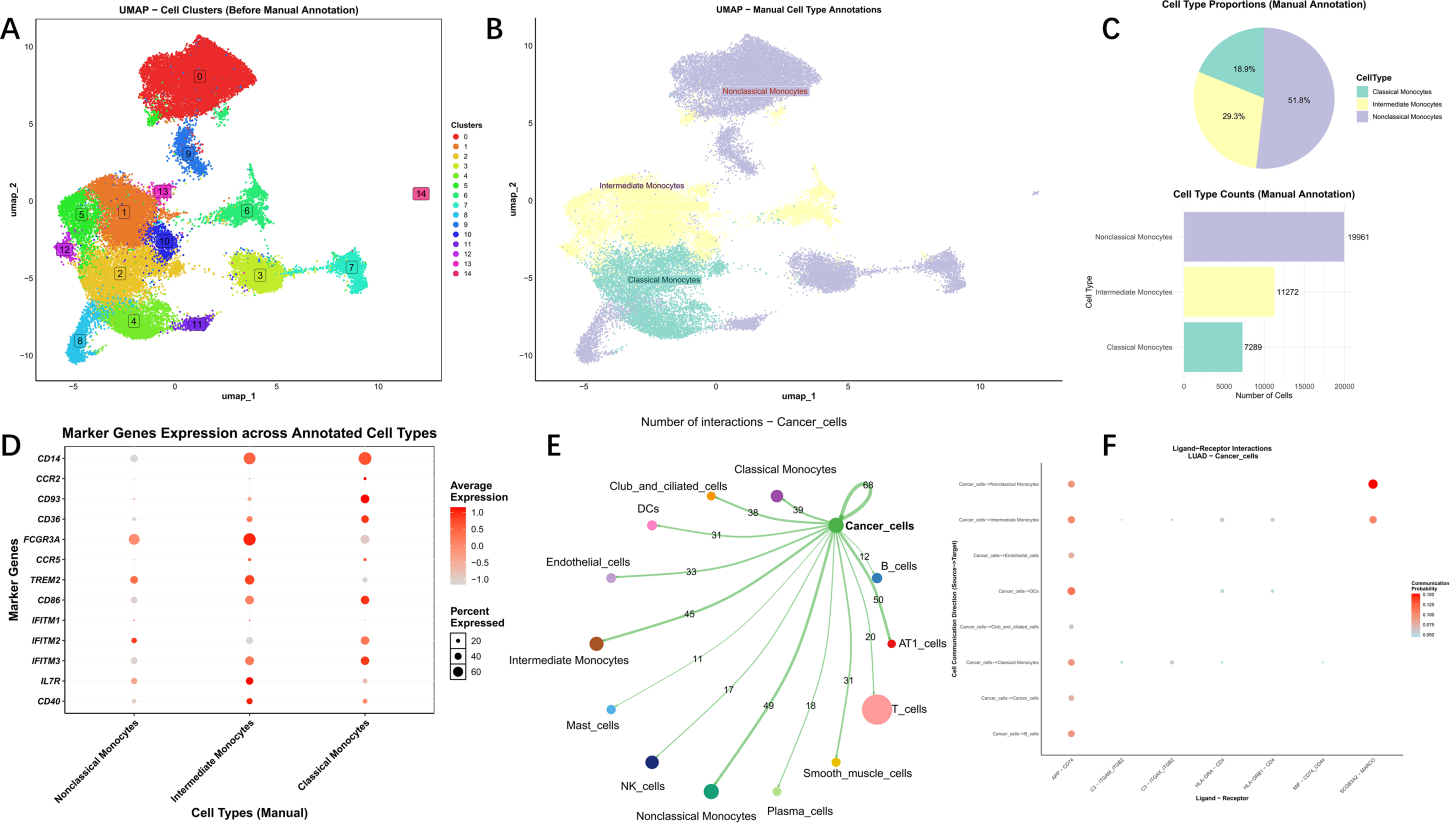
Analysis of Monocyte subpopulations and cell communication. **(A)** Distribution of monocyte subtypes in UMAP clusters; **(B)** UMAP clusters of monocyte subtypes in LUAD; **(C)** Cell type proportions; **(D)**Marker gene expression profiles; **(E)** The interaction network of CellChat for cancer cells; **(F)** Dot plot for the enrichment of ligand-receptor pathways for cancer cells.

We then extracted the monocyte subpopulation and applied the same pipeline for dimensionality reduction, clustering, and batch effect correction. After initial processing and further dimensionality reduction of the principal components via UMAP, three distinct monocyte subsets were identified: classical, intermediate, and non-classical monocytes (**Figures 9A, B**). The non-classical monocyte subset was the most prevalent (**Figure 9C**). Marker genes for each subgroup are shown in **Figure 9D**.

**Figure 10:**
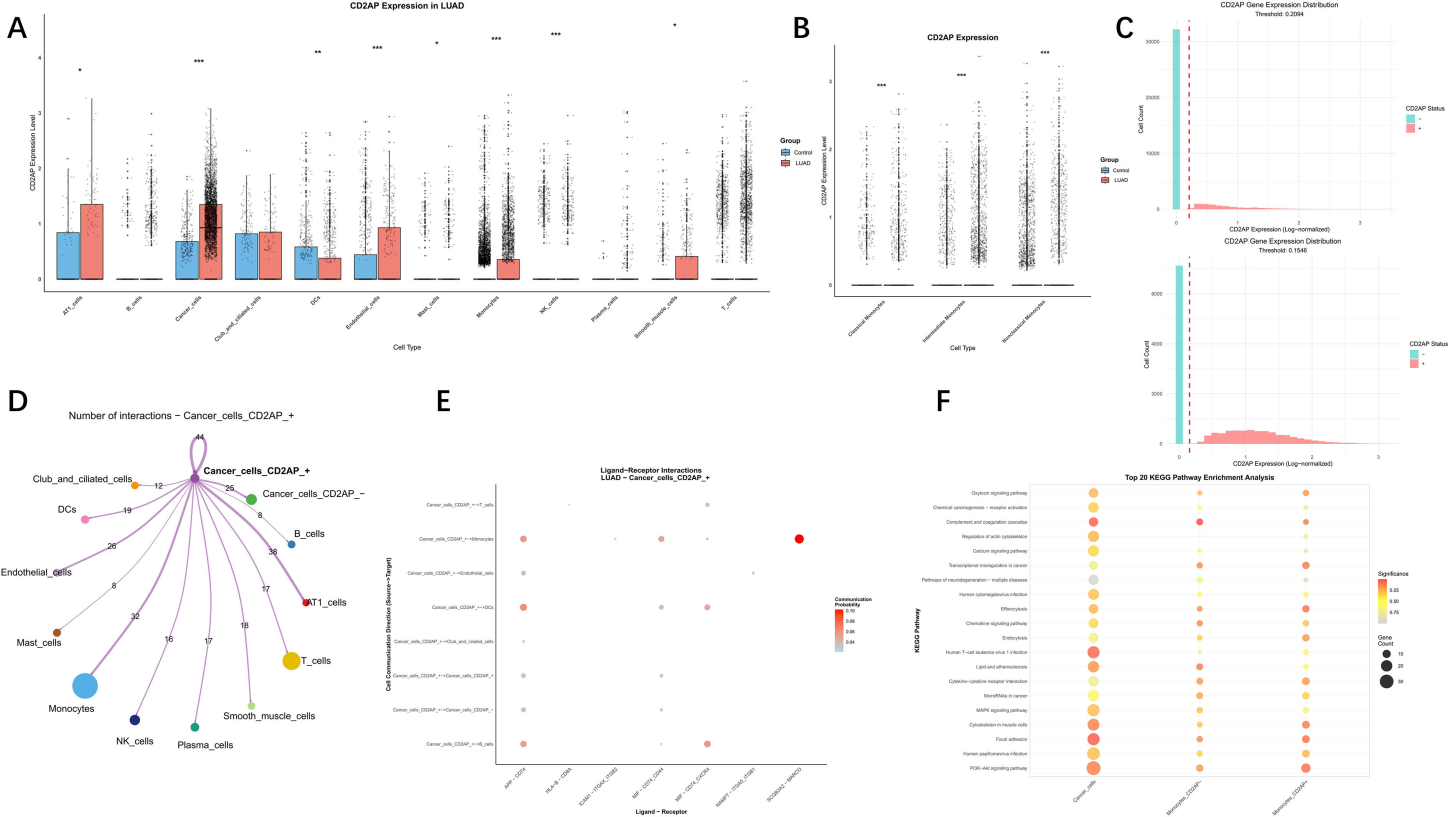
Exploration of *CD2AP* functions. **(A)***CD2AP* gene expression across all cell types; **(B)***CD2AP* gene expression in monocyte subtypes; **(C)** Expression distribution of *CD2AP* in cancer cells and monocytes; **(D)** Cellular interaction network of *CD2AP*+ cancer cells LUAD; **(E)** Dot plot for the enrichment of ligand-receptor pathways; **(F)** KEGG pathway enrichment analysis for *CD2AP*+ and *CD2AP*- monocytes.

To delve deeper into the roots of numerical and functional changes in monocytes, we utilised CellChat to decipher the intercellular communication network. Visualisation revealed that all three monocyte subsets exhibited strong interactions with tumour cells compared to other cell types (**Figure 9E**), suggesting a close biological relationship between them. By visualising the ligand-receptor signalling networks underlying this communication, we identified that interactions between tumour cells and both non-classical and intermediate monocytes were primarily mediated by the APP-CD74 and SCGB3A2-MARCO signalling axes (**Figure 9F**). Subsequently, we investigated the expression pattern of the target gene *CD2AP* across different cell subtypes in LUAD. The results demonstrated that *CD2AP* expression was significantly upregulated in monocytes, endothelial cells, and NK cells within tumour tissues compared to their counterparts in normal tissues (**Figure 10A**). Importantly, within the monocyte population, all three subsets exhibited significantly higher *CD2AP* expression in LUAD (**Figure 10B**). These findings indicate that the positive correlation observed in bulk RNA sequencing data is likely driven, at least in part, by the intrinsically high expression of *CD2AP* within the infiltrating immune cells themselves, in addition to tumour cells. To further explore the role of *CD2AP* in tumour cells and monocytes, we stratified these two cell types into *CD2AP*+ and *CD2AP*- subgroups using expression thresholds of 0.2094 and 0.1546, respectively (**Figure 10C**). Cell communication analysis indicated strong interactions between *CD2AP*+ cancer cells and monocytes, predominantly mediated by the APP-CD74, MIF-CD74_CD44, and SCGB3A2-MARCO pathways (**Figures 10D, E**). Finally, KEGG pathway enrichment analysis revealed that *CD2AP*+ Monocytes were differentially enriched in pathways such as the PI3K-Akt signalling pathway, focal adhesion, and efferocytosis (**Figure 10F**).

### Drug sensitivity analysis and molecular docking for CD2AP

**Figure 11:**
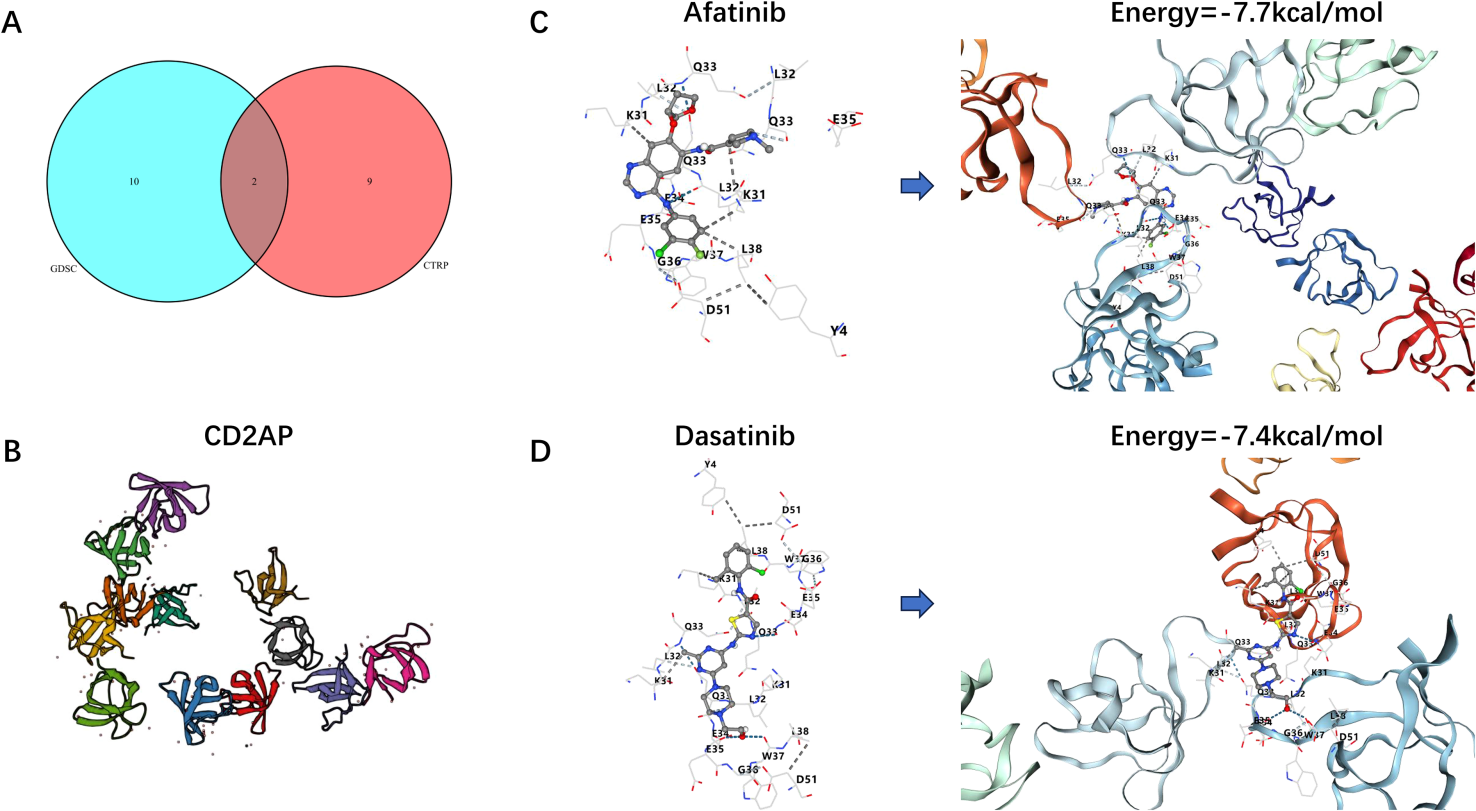
Drug sensitivity analysis and molecular docking. **(A)** Venn plot indicating the potential targeted compounds. **(B)** The structures of CD2AP. **(C)** Molecular docking between afatinib and CD2AP. **(D)** Molecular docking between dasatinib and CD2AP.

As *CD2AP* was identified as the key regulator and the enrichment of its mRNA and protein was predicted to indicate poor clinical outcomes, it could be regarded as a potential therapeutic target for LUAD. The drugs correlated with *CD2AP* were obtained from the GDSC (**Supplementary Table S6**) and CTRP (**Supplementary Table S7**) databases, while the potential targeting drugs were screened out using the criteria of FDR < 0.05 and a correlation coefficient (R) < 0. In total, 12 and 11 types of compounds were identified in the CDSC and CTRP databases, respectively. Among them, two drugs (afatinib and dasatinib) revealed compelling evidence for targeting *CD2AP*, as they were identified as intersections of the two databases (**Figure 11A**).

To assess the affinity of these two drug candidates for the CD2AP protein, we performed molecular docking analysis, and the structures of CD2AP are shown in **Figure 11B**. CB-Dock2 was used to determine the binding postures and interactions of two drug candidates with CD2AP and to calculate the corresponding binding energies. The results show that both afatinib and dasatinib bind CD2AP through visible hydrogen bonding and strong electrostatic interactions. In addition, the hydrophobic pocket of CD2AP was successfully occupied by two drug candidates; meanwhile, afatinib and dasatinib had low binding energies of -7.7 and -7.4 kcal/mol, indicating a highly stable binding (**Figures 11C, D**).

### Experimental validation

**Figure 12:**
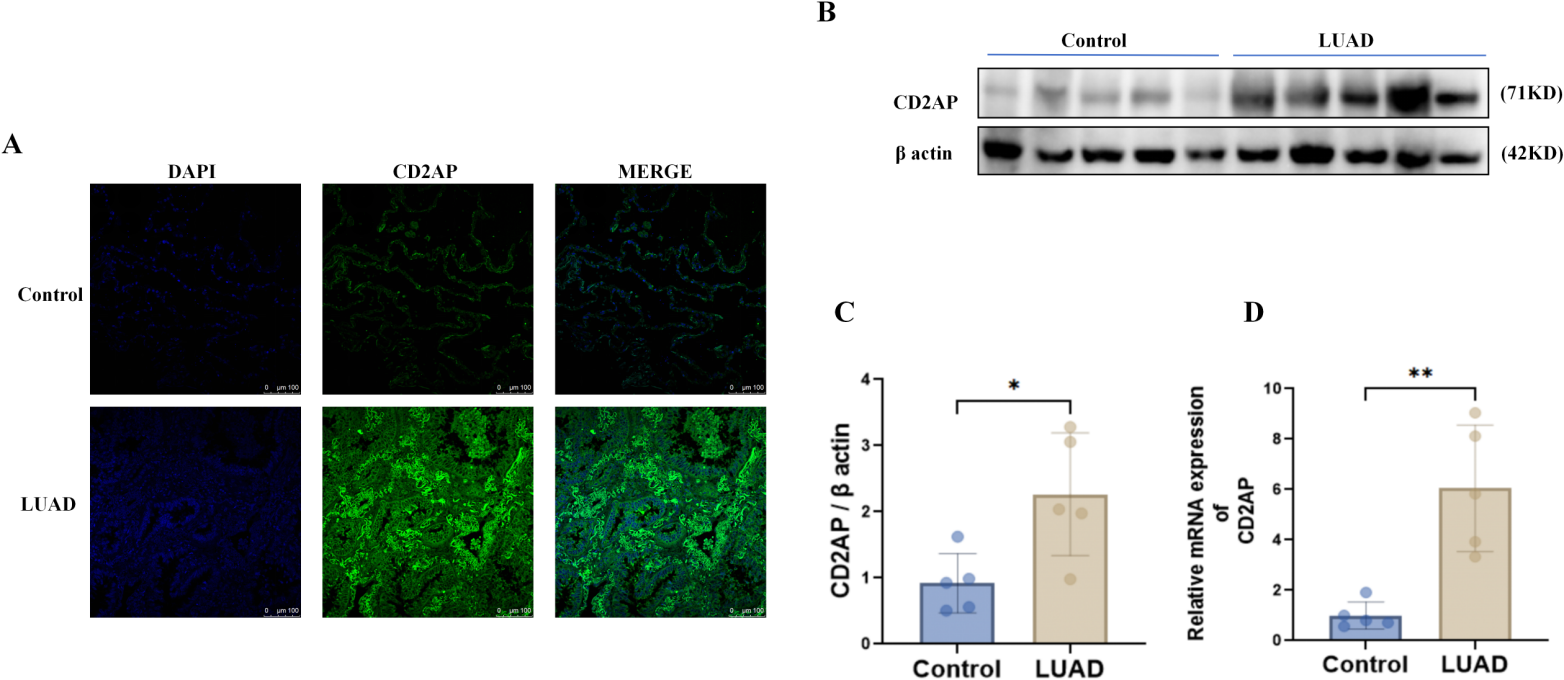
Histological experiments to validate the expression of CD2AP. **(A)** Validation of CD2AP expression in cancer and adjacent tissues by immunofluorescence (n=3 biologically independent samples); **(B)** Determine CD2AP protein levels using Western blot analysis; **(C)** Statistical chart for WB, data are presented as mean ± SD (n=5 biologically independent samples). *P < 0.05; **(D)** Results of rt-qPCR for 5 controls vs. 5 LUADs, data are presented as mean ± SD (n=5 biologically independent samples). **P < 0.01.

We initially employed tissue immunofluorescence, which revealed a significant increase in CD2AP protein levels in LUAD tissues. The intense CD2AP fluorescence was predominantly localised to the cytoplasm rather than the nucleus (**Figure 12A**). Subsequently, we assessed CD2AP expression in paired tumour and adjacent normal tissues from five LUAD patients using Western Blot (WB). The results confirmed that CD2AP protein levels were significantly elevated in tumour tissues (**Figure 12B**), with the quantitative analysis shown in the corresponding histogram (*P* < 0.05, **Figure 12C**). Consistent with these findings, RT-qPCR analysis demonstrated a marked upregulation of *CD2AP* mRNA expression in tumour samples (**Figure 12D**).

**Figure 13:**
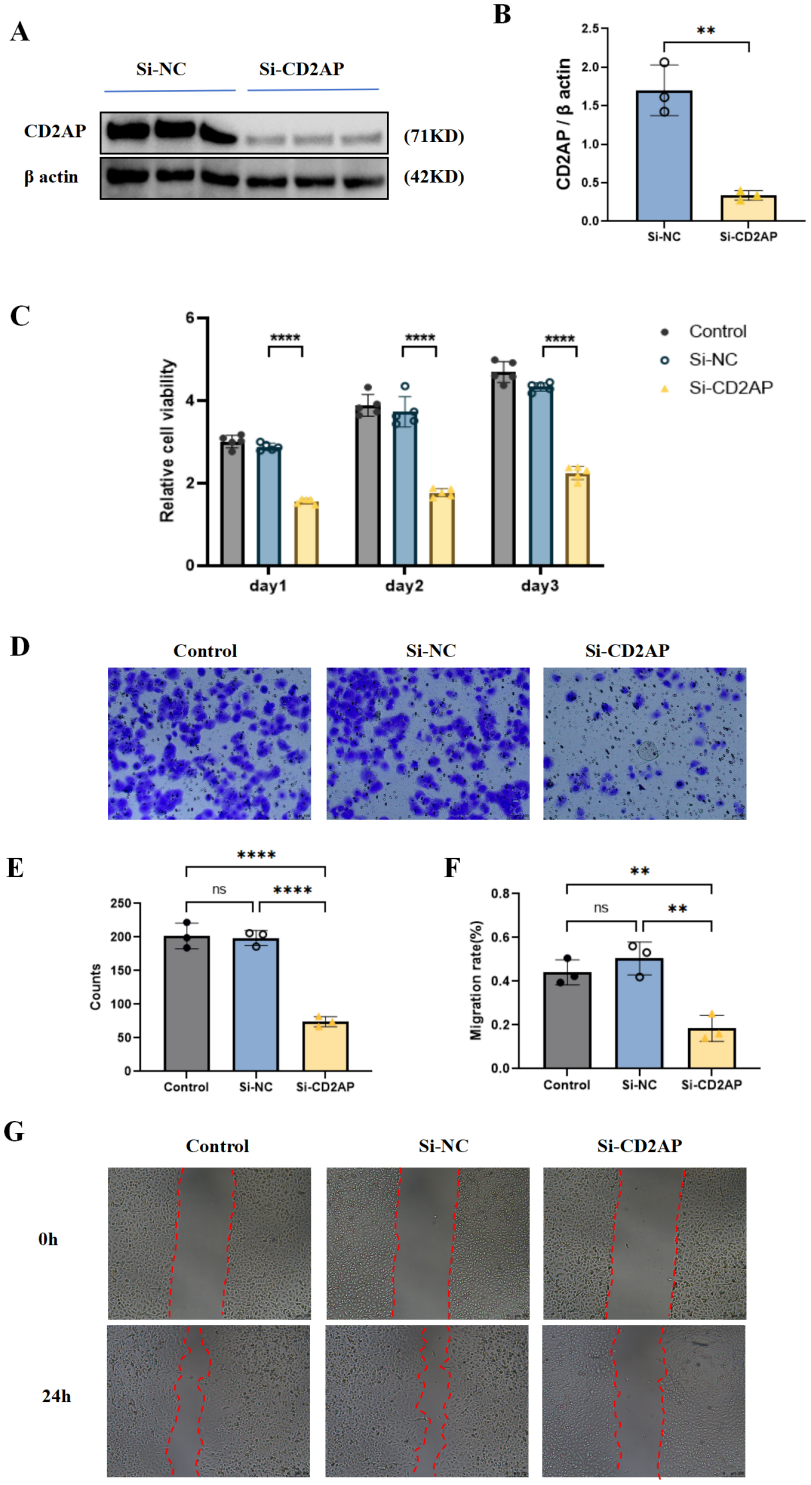
Cell experiments to validate the function of CD2AP. **(A)** WB to show the knockdown efficiency of CD2AP; **(B)** Statistical chart for WB, data are presented as mean ± SD (n=3 biologically independent samples). **P < 0.01; **(C)** Proliferative capacity of A549 cells by CCK-8 assay, data are presented as mean ± SD (n=3 technical replicates per group, representative of three independent experiments); **(D)** Cell migration capabilities by transwell; **(E)** Statistical chart for transwell counts, data are presented as mean ± SD (n=3 biologically independent samples). ****P < 0.0001; **(F)** Cell migration capabilities by wound healing assays; **(G)** Statistical chart for wound healing assays, data are presented as mean ± SD (n=3 biologically independent samples). **P < 0.01.

We then proceeded to functional validation in cell-based models. Prior staging analysis suggested *CD2AP* likely functions as an oncogene and is highly expressed in tumour cells. To investigate this, we utilised the A549 cell line for further experiments. *CD2AP* expression was effectively knocked down using specific siRNA (**Figures 13A, B**). A CCK-8 assay revealed that the proliferative capacity of A549 cells transfected with si-*CD2AP* was significantly impaired compared to controls (**Figure 13C**). Furthermore, we assessed cell migration capabilities using transwell and wound healing assays. The results demonstrated that silencing *CD2AP* led to a significant reduction in the migratory ability of A549 cells (**Figures 13D–G**).

## Discussion

In this study, we undertook a systematic investigation to delineate the prognostic landscape of ubiquitination-related regulators in LUAD. Our approach was two-pronged: first, we developed and validated a robust five-gene prognostic signature for stratifying LUAD patients, which acknowledges the complex, polygenic nature of cancer progression. Second, and more importantly, we delved deeper into this model to identify a pivotal core molecule–CD2AP. This strategy, progressing from a multigene ensemble to a singular key driver, enabled us to bridge the prognostic power of a composite model with focussed, target-specific mechanistic exploration and therapeutic feasibility. Through this robust screening pipeline integrating differential expression, survival analysis, and proteomic validation, CD2AP emerged as a prominent candidate. Its consistent overexpression at both the mRNA and protein levels, coupled with a strong association with poor survival across multiple cohorts, underscores its potential as a novel oncoprotein in LUAD worthy of in-depth functional and translational investigation.

We identified *CD2AP* as a key molecular, as its mRNA and protein levels were elevated in tumour tissues; meanwhile, higher expression of *CD2AP* predicted poorer clinical outcomes across multiple databases. The expression level of *CD2AP* was proven to be associated with TMBs, immune cell infiltrations, and tumour T stages in subsequent analysis. The functional analyses revealed that *CD2AP* was closely associated with protein and amino acid metabolism, as well as the corresponding diseases. The single-cell analysis revealed that *CD2AP* was primarily enriched in mononuclear macrophages, highlighting its critical role in mediating intercellular communication between tumour cells and monocyte subsets, thereby illuminating a previously unrecognised mechanism of tumour-stroma crosstalk. Moreover, we conducted a drug sensitivity analysis and screened out two drugs (afatinib and dasatinib) that could be utilised for *CD2AP* highly expressed LUAD through molecular docking analysis. Our histological and cytological experiments demonstrated that both the protein and mRNA expression levels of *CD2AP* were significantly elevated in tumour tissues compared to adjacent non-cancerous tissues. Furthermore, knockdown of *CD2AP* in A549 cells markedly attenuated their proliferative and migratory capacities.

CD2AP proteins are closely linked to the process of ubiquitination. Ubiquitination involves a series of reactions catalysed by ubiquitin-activating enzyme E1, ubiquitin-conjugating enzyme E2, and ubiquitin-conjugating enzyme E3, through which ubiquitin molecules are covalently bound to target proteins, thereby affecting the stability, activity, or intracellular localisation of these proteins (24). This process is not only involved in protein degradation but also plays an important role in the regulation of cellular functions, such as signal transduction, DNA damage repair, and immune response, etc. (25). The CD2AP-associated ubiquitination process plays important roles in a variety of diseases, particularly in AD. CD2AP is a risk factor for AD, and its abnormalities may be involved in the disease at multiple levels, including affecting the transport and degradation processes of amyloid precursor protein (APP) (26), which influences Aβ production and deposition; participating in Tau protein-mediated neurotoxicity (27); interfering with synaptic function and vesicle release; and affecting the integrity of the blood-brain barrier (28). Consistent with our results, we further demonstrated that *CD2AP*-expressing cancer cells mediate intercellular communication with monocytes primarily via the APP-CD74 pathway. In the nervous system, CD2AP co-localises with Rab5 and is involved in the regulation of endosome morphology, as well as endosome-to-lysosome transport processes (29). In addition, CD2AP, as one of the essential proteins in podocytes, possesses a unique SH3 structural domain that enables it to interact with other protein molecules in podocytes, such as α-actinin-4 and Podocin. Together, these proteins maintain the normal morphology and physiological function of the podocyte slit diaphragm (30). Experiments have confirmed that decreased expression, deletion, and damage of *CD2AP* can impair the podocyte slit diaphragm, cause cytoskeletal disruption of podocytes, and affect the permeability of the filtration membrane, resulting in proteinuria (31). In diabetic kidney disease (DKD), high glucose-associated damage factors lead to abnormal foot cell proteasome function, altered ubiquitin ligase expression levels, and increased deubiquitinase activity, which in turn result in the accumulation of damaging proteins or the aberrant degradation of protective proteins, thereby promoting podocyte damage (32, 33). These findings suggest that the abnormal expression and dysfunction of *CD2AP* in renal diseases may be related to the ubiquitination process, which affects the structure and function of podocytes and thus contributes to the development of these diseases.

However, the specific role of *CD2AP* in malignancies, especially in lung cancers, has been less elucidated. Studies have shown that *CD2AP* is involved in regulating cytoskeletal and endosomal-lysosomal pathways, which affects the proliferation, migration, and invasive ability of tumour cells (34). In glioblastoma, *CD2AP* promotes tumour progression through TRIM5-mediated NF-kB signalling (35). In addition, abnormalities in *CD2AP* expression and function may be associated with tumour cell invasiveness and drug resistance (36). However, another study proved that *CD2AP* inhibited tumour metastasis and played an anti-tumour role in gastric cancer (37). Chen et al. found that *CD2AP* was down-regulated and predicted a better prognosis in renal clear cell carcinoma (38), a conclusion consistent with the results of our pan-cancer analysis (CCRCC in **Figure 4C**, KIRC in **Supplementary Figure S2A**). Therefore, the role of *CD2AP* in various types of tumours is inconsistent. Our study found that *CD2AP* mRNA was upregulated in tumour tissues, and its higher expression predicted a poor prognosis for LUAD patients, suggesting that it functions as a cancer-promoting gene. Meanwhile, the enrichment of CD2AP protein was also associated with lower survival rates in LUAD patients, suggesting that targeting CD2AP protein may be a novel therapeutic method. Interestingly, the *CD2AP* mRNA level was not enriched, while the CD2AP protein level was somehow down-regulated in lung squamous carcinoma.

There is growing evidence that the ubiquitination process plays a key regulatory role in both intrinsic and adaptive immune responses by modulating the function of various cell types within the immune system (39). Consistent with our bulk tissue analysis, single-cell data confirmed a strong link between *CD2AP* and monocytes/macrophages, revealing that these immune cells are a significant source of *CD2AP* expression in the TME. Based on the strong correlation and the finding that monocytes themselves express high levels of *CD2AP* in the TME (**Figures 10A, B**), we hypothesise that the *CD2AP*-high ecosystem–comprising both tumour cells and immune cell–may foster a pro-tumoural environment. The functional role of *CD2AP* within monocytes/macrophages and how it contributes to tumour progression remain to be fully elucidated and represent an important direction for future research.

Currently, ubiquitination-based antitumour-targeted therapies have shown initial success. Compared with traditional antitumour drugs, ubiquitination component inhibitors exhibit specificity in recognising substrates, which can effectively reduce nonspecific side effects and the resistance of cancer cells (40). However, despite the promising potential of ubiquitination as a new target for tumour therapy, many antitumour studies targeting ubiquitination are still in the early stages. Our study provides insights into the development of novel, ubiquitination-related targeted drugs. The study of the specific recognition properties of UBDs and their regulation of various biological processes is the basis for our investigation of ubiquitin signalling networks (41). UBDs are key nodes of ubiquitin regulation, and their regulatory roles in cancer and immunodeficiency diseases have received increasing attention, making them highly likely to become next-generation therapeutic targets. In this study, molecular docking analysis identified two previously used drugs that can be repurposed for a group of patients with *CD2AP* highly expressed. Afatinib, the second-generation drug of tyrosine-kinase inhibitors (TKIs), is commonly used for those with EGFR-mutated NSCLCs (42). Our study demonstrated the indications of afatinib for those with high expression of *CD2AP* and provided new strategies for treating LUAD. It is plausible that in *CD2AP*-high tumours, the oncogenic activity is partly dependent on a synergistic interplay between EGFR signalling and *CD2AP*-mediated scaffolding and endocytosis. Afatinib, by simultaneously inhibiting EGFR and potentially disrupting CD2AP function, could thereby exert a more potent effect in this molecular subset. As a second-generation tyrosine kinase inhibitor, dasatinib is commonly used in the treatment of chronic myelogenous leukaemia (CML) and Philadelphia chromosome-positive acute lymphoblastic leukaemia (ALL) (43). When combined with other drugs, dasatinib can inhibit the growth of cancer cells in various solid tumours, including lung cancer; however, the specific mechanisms are diverse (44). Dasatinib is a broad-spectrum kinase inhibitor targeting SRC and other families (45), and the connection is equally compelling. *CD2AP* has been reported to interact with proteins in SRC-related pathways, which are pivotal for cell migration and invasion. The ability of dasatinib to inhibit SRC signalling is an established mechanism for its anti-metastatic effects. Our docking prediction raises the hypothesis that dasatinib’s efficacy might be augmented in *CD2AP*-high tumours by concurrently inhibiting SRC kinases and disrupting *CD2AP*-dependent invasive structures.

Although our in silico molecular docking analysis indicates promising binding potential of afatinib and dasatinib to CD2AP, it is crucial to acknowledge the inherent limitations of this approach. The calculated binding energies suggest moderate affinity, and computational simulations cannot fully replicate the complex intracellular environment, protein-protein interactions, or off-target effects. Therefore, while these findings provide a compelling rationale for drug repurposing, the actual binding specificity and *in vivo* inhibitory efficacy of these drugs against *CD2AP* require further experimental validation through *in vitro* binding assays and functional studies in preclinical models.

Despite the comprehensive nature of our study, several limitations should be acknowledged. First, the cohort of clinical samples used for experimental validation (qPCR, Western Blot, and immunofluorescence) was relatively small (N = 13), although it encompassed patients across different disease stages. Second, while our *in vitro* functional assays demonstrated that *CD2AP* silencing suppresses proliferation and migration in A549 cells, the lack of *in vivo* validation in animal models means the oncogenic role of *CD2AP* in a complex physiological context remains to be fully confirmed. Third, although our bioinformatic analyses (such as KEGG and ssGSEA) implicated *CD2AP* in pathways like oxidative phosphorylation, amino acid metabolism, and the TGF-β pathway, the precise downstream signalling mechanisms through which *CD2AP* exerts its pro-tumorigenic effects and modulates the immune microenvironment are not fully elucidated and require further mechanistic investigation. Finally, the drug candidates (afatinib and dasatinib) identified through computational docking, while promising, are predictions that necessitate subsequent *in vitro* and *in vivo* experimental validation to confirm their efficacy in targeting *CD2AP*-high LUAD. Addressing these limitations will be the focus of our future work.

## Conclusion

In summary, our study systematically delineates the pivotal role of the ubiquitin-proteasome system in LUAD pathogenesis. We have developed and validated a robust five-gene ubiquitination-related signature that effectively stratifies LUAD patients into distinct prognostic groups. Central to this signature is *CD2AP*, which we identified as a novel oncoprotein consistently overexpressed at both the mRNA and protein levels in LUAD. Its elevated expression is a powerful indicator of poor clinical outcomes, advanced tumour stage, and an immunosuppressive microenvironment characterised by specific monocyte/macrophage infiltration.

Through the integration of multi-omics data and functional experiments, we demonstrated that *CD2AP* promotes tumour cell proliferation and migration. Furthermore, scRNA-seq analysis revealed its critical role in mediating intercellular communication between tumour cells and monocyte subsets, thereby illuminating a previously unrecognised mechanism of tumour-stroma crosstalk. Translating these findings into therapeutic potential, we identified afatinib and dasatinib as promising candidate drugs that exhibit high binding affinity for the CD2AP protein. Our work not only establishes *CD2AP* as a key prognostic biomarker and therapeutic target but also provides a compelling rationale for repurposing existing tyrosine kinase inhibitors to treat patients with LUAD and *CD2AP*-high tumours, ultimately offering a novel precision medicine strategy for this challenging disease.

## Data Availability

The original contributions presented in the study are included in the article/**Supplementary Material**. Further inquiries can be directed to the corresponding author.

## Supplementary material

Supplementary Figure 1 Validation of the risk signature in an external GEO cohort. **(A)** The distribution of risk scores for each individual in the validation cohort. **(B)** The PCA plot for patients. **(C)** The t-SNE analysis. **(D)** Survival status for patients (low-risk: left of the dotted line; high-risk: right of the dotted line). **(E)** Kaplan–Meier curves for the OS in two risk groups. **(F)** Time-dependent ROC curves.

Supplementary Figure 2 Exploration of *CD2AP* functions. **(A)** Pan-cancer analysis comparing *CD2AP* mRNA levels in cancer and normal tissues. **(B)** Meta-analysis to compare *CD2AP* expression levels between normal and LUAD tissues. **(C)** Meta-analysis for assessing the prognostic value of *CD2AP* in LUAD. **(D)** CD2AP-centric PPI network (interaction score was set at 0.4).

Supplementary Figure 3 The correlations between *CD2AP* expression levels and clinical features.

Supplementary Figure 4 Correlation between *CD2AP* expression and pathway enrichment scores derived from single-sample GSEA (ssGSEA).

Supplementary Figure 5 Preprocessing and filtering of single-cell RNA sequencing data **(A)** the quality control metrics of sequencing data, number of detected features (nFeature_RNA), the total RNA count (nCount_RNA), the percentage of mitochondrial genes (percent.mt); **(B)** the percentage of haemoglobin genes; **(C)** PCA analysis before calibration; **(D)** Scatter plot after batch effect correction.

Supplementary Table 1 The list of the 807 ubiquitination-related genes.

Supplementary Table 2 Clinicopathological characteristics of the 13 LUAD patients in the experimental cohort.

Supplementary Table 3 Comparison of the expression levels of the ubiquitination-related genes between normal (normMean) and tumour (tumorMean) tissues.

Supplementary Table 4 The prognostic values of the ubiquitination-related genes.

Supplementary Table 5 Marker genes for each cell in LUAD.

Supplementary Table 6 Drugs correlated with *CD2AP* obtained from the GDSC database.

Supplementary Table 7 Drugs correlated with *CD2AP* obtained from the CTRP database.

## References

[1] Testa U Castelli G Pelosi E . Lung cancers: molecular characterization, clonal heterogeneity and evolution, and cancer stem cells. Cancers. (2018) 10:248. doi: 10.3390/cancers10080248, PMID: 30060526 PMC6116004

[2] Asamura H Goya T Koshiishi Y Sohara Y Eguchi K Mori M . A Japanese Lung Cancer Registry study: prognosis of 13,010 resected lung cancers. J Thorac Oncol. (2008) 3:46–52. doi: 10.1097/JTO.0b013e31815e8577, PMID: 18166840

[3] Spella M Stathopoulos G . Immune resistance in lung adenocarcinoma. Cancers. (2021) 13:384. doi: 10.3390/cancers13030384, PMID: 33494181 PMC7864325

[4] Swatek K Komander D . Ubiquitin modifications. Cell Res. (2016) 26:399–422. doi: 10.1038/cr.2016.39, PMID: 27012465 PMC4822133

[5] Sun T Liu Z Yang Q . The role of ubiquitination and deubiquitination in cancer metabolism. Mol Cancer. (2020) 19:146. doi: 10.1186/s12943-020-01262-x, PMID: 33004065 PMC7529510

[6] Pfoh R Lacdao I Saridakis V . Deubiquitinases and the new therapeutic opportunities offered to cancer. Endocrine-Related Cancer. (2015) 22:T35–54. doi: 10.1530/ERC-14-0516, PMID: 25605410 PMC4304536

[7] Skaar J Pagan J Pagano M . SCF ubiquitin ligase-targeted therapies. Nat Rev Drug Discov. (2014) 13:889–903. doi: 10.1038/nrd4432, PMID: 25394868 PMC4410837

[8] Fennell L Rahighi S Ikeda F . Linear ubiquitin chain-binding domains. FEBS J. (2018) 285:2746–61. doi: 10.1111/febs.14478, PMID: 29679476

[9] Dikic I Wakatsuki S Walters K . Ubiquitin-binding domains - from structures to functions. Nat Rev Mol Cell Biol. (2009) 10:659–71. doi: 10.1038/nrm2767, PMID: 19773779 PMC7359374

[10] Tao QQ Chen YC Wu ZY . The role of CD2AP in the pathogenesis of alzheimer’s disease. Aging Dis. (2019) 10:901–7. doi: 10.14336/AD.2018.1025, PMID: 31440393 PMC6675523

[11] Camacho J Rabano A Marazuela P Bonaterra-Pastra A Serna G Moline T . Association of CD2AP neuronal deposits with Braak neurofibrillary stage in Alzheimer’s disease. Brain Pathol. (2022) 32:e13016. doi: 10.1111/bpa.13016, PMID: 34514662 PMC8713526

[12] Feng D . Phosphorylation of key podocyte proteins and the association with proteinuric kidney disease. Am J Physiol Renal Physiol. (2020) 319:F284–F91. doi: 10.1152/ajprenal.00002.2020, PMID: 32686524 PMC7528399

[13] Charisis S Lin H Ray R Joehanes R Beiser AS Levy D . Obesity impacts the expression of Alzheimer’s disease-related genes: The Framingham Heart Study. Alzheimers Dement. (2023) 19:3496–505. doi: 10.1002/alz.12954, PMID: 36811231 PMC10435662

[14] Shen T Cai L Liu Y Li S Gan W Li X . Ube2v1-mediated ubiquitination and degradation of Sirt1 promotes metastasis of colorectal cancer by epigenetically suppressing autophagy. J Hematol Oncol. (2018) 11:95. doi: 10.1186/s13045-018-0638-9, PMID: 30016968 PMC6050692

[15] Duhamel S Goyette M Thibault M Filion D Gaboury L Côté J . The E3 ubiquitin ligase hectD1 suppresses EMT and metastasis by targeting the +TIP ACF7 for degradation. Cell Rep. (2018) 22:1016–30. doi: 10.1016/j.celrep.2017.12.096, PMID: 29386124

[16] He M Zhou Z Wu G Chen Q Wan Y . Emerging role of DUBs in tumor metastasis and apoptosis: Therapeutic implication. Pharmacol Ther. (2017) 177:96–107. doi: 10.1016/j.pharmthera.2017.03.001, PMID: 28279784 PMC5565705

[17] Wang J Song X Wei M Qin L Zhu Q Wang S . PCAS: an integrated tool for multi-dimensional cancer research utilizing clinical proteomic tumor analysis consortium data. Int J Mol Sci. (2024) 25:6690. doi: 10.3390/ijms25126690, PMID: 38928396 PMC11203781

[18] Ye Y Dai Q Qi H . A novel defined pyroptosis-related gene signature for predicting the prognosis of ovarian cancer. Cell Death Discov. (2021) 7:71. doi: 10.1038/s41420-021-00451-x, PMID: 33828074 PMC8026591

[19] Li T Fu J Zeng Z Cohen D Li J Chen Q . TIMER2.0 for analysis of tumor-infiltrating immune cells. Nucleic Acids Res. (2020) 48:W509–W14. doi: 10.1093/nar/gkaa407, PMID: 32442275 PMC7319575

[20] Bischoff P Trinks A Obermayer B Pett JP Wiederspahn J Uhlitz F . Single-cell RNA sequencing reveals distinct tumor microenvironmental patterns in lung adenocarcinoma. Oncogene. (2021) 40:6748–58. doi: 10.1038/s41388-021-02054-3, PMID: 34663877 PMC8677623

[21] Liu CJ Hu FF Xie GY Miao YR Li XW Zeng Y . GSCA: an integrated platform for gene set cancer analysis at genomic, pharmacogenomic and immunogenomic levels. Brief Bioinform. (2023) 24:bbac558. doi: 10.1093/bib/bbac558, PMID: 36549921

[22] Liu Y Yang X Gan J Chen S Xiao ZX Cao Y . CB-Dock2: improved protein-ligand blind docking by integrating cavity detection, docking and homologous template fitting. Nucleic Acids Res. (2022) 50:W159–W64. doi: 10.1093/nar/gkac394, PMID: 35609983 PMC9252749

[23] Cai L Lin S Girard L Zhou Y Yang L Ci B . LCE: an open web portal to explore gene expression and clinical associations in lung cancer. Oncogene. (2019) 38:2551–64. doi: 10.1038/s41388-018-0588-2, PMID: 30532070 PMC6477796

[24] Collins G Goldberg A . The logic of the 26S proteasome. Cell. (2017) 169:792–806. doi: 10.1016/j.cell.2017.04.023, PMID: 28525752 PMC5609836

[25] Sheng X Xia Z Yang H Hu R . The ubiquitin codes in cellular stress responses. Protein Cell. (2024) 15:157–90. doi: 10.1093/procel/pwad045, PMID: 37470788 PMC10903993

[26] Liao F Jiang H Srivatsan S Xiao Q Lefton KB Yamada K . Effects of CD2-associated protein deficiency on amyloid-beta in neuroblastoma cells and in an APP transgenic mouse model. Mol Neurodegener. (2015) 10:12. doi: 10.1186/s13024-015-0006-y, PMID: 25887956 PMC4374406

[27] Szabo MP Mishra S Knupp A Young JE . The role of Alzheimer’s disease risk genes in endolysosomal pathways. Neurobiol Dis. (2022) 162:105576. doi: 10.1016/j.nbd.2021.105576, PMID: 34871734 PMC9071255

[28] Ojelade SA Lee TV Giagtzoglou N Yu L Ugur B Li Y . cindr, the drosophila homolog of the CD2AP alzheimer’s disease risk gene, is required for synaptic transmission and proteostasis. Cell Rep. (2019) 28:1799–813.e5. doi: 10.1016/j.celrep.2019.07.041, PMID: 31412248 PMC6703184

[29] Furusawa K Takasugi T Chiu YW Hori Y Tomita T Fukuda M . CD2-associated protein (CD2AP) overexpression accelerates amyloid precursor protein (APP) transfer from early endosomes to the lysosomal degradation pathway. J Biol Chem. (2019) 294:10886–99. doi: 10.1074/jbc.RA118.005385, PMID: 31138646 PMC6635452

[30] Fan Q Xing Y Ding J Guan N Zhang J . The relationship among nephrin, podocin, CD2AP, and alpha-actinin might not be a true ‘interaction’ in podocyte. Kidney Int. (2006) 69:1207–15. doi: 10.1038/sj.ki.5000245, PMID: 16501493

[31] Lepa C Moller-Kerutt A Stolting M Picciotto C Eddy ML Butt E . LIM and SH3 protein 1 (LASP-1): A novel link between the slit membrane and actin cytoskeleton dynamics in podocytes. FASEB J. (2020) 34:5453–64. doi: 10.1096/fj.201901443R, PMID: 32086849

[32] Chung JJ Goldstein L Chen YJ Lee J Webster JD Roose-Girma M . Single-cell transcriptome profiling of the kidney glomerulus identifies key cell types and reactions to injury. J Am Soc Nephrol. (2020) 31:2341–54. doi: 10.1681/ASN.2020020220, PMID: 32651223 PMC7609001

[33] Li O Ma Q Li F Cai GY Chen XM Hong Q . Progress of small ubiquitin-related modifiers in kidney diseases. Chin Med J (Engl). (2019) 132:466–73. doi: 10.1097/CM9.0000000000000094, PMID: 30707172 PMC6595721

[34] Kurilla A Laszlo L Takacs T Tilajka A Lukacs L Novak J . Studying the association of TKS4 and CD2AP scaffold proteins and their implications in the partial epithelial-mesenchymal transition (EMT) process. Int J Mol Sci. (2023) 24:15136. doi: 10.3390/ijms242015136, PMID: 37894817 PMC10606890

[35] Zhang L He J Zhao W Zhou Y Li J Li S . CD2AP promotes the progression of glioblastoma multiforme via TRIM5-mediated NF-kB signaling. Cell Death Dis. (2024) 15:722. doi: 10.1038/s41419-024-07094-7, PMID: 39353894 PMC11445578

[36] Zhang K Zhu Z Zhou J Shi M Wang N Yu F . Disulfidptosis-related gene expression reflects the prognosis of drug-resistant cancer patients and inhibition of MYH9 reverses sorafenib resistance. Transl Oncol. (2024) 49:102091. doi: 10.1016/j.tranon.2024.102091, PMID: 39146597 PMC11375144

[37] Xie W Chen C Han Z Huang J Liu X Chen H . CD2AP inhibits metastasis in gastric cancer by promoting cellular adhesion and cytoskeleton assembly. Mol Carcinog. (2020) 59:339–52. doi: 10.1002/mc.23158, PMID: 31989722 PMC7078920

[38] Chen C Xu J Zhang JX Chen LY Wei YA Zhang WM . CD2AP is a potential prognostic biomarker of renal clear cell carcinoma. Cancer Med. (2024) 13:e7055. doi: 10.1002/cam4.7055, PMID: 38457255 PMC10923042

[39] Fujita Y Tinoco R Li Y Senft D Ronai Z . Ubiquitin ligases in cancer immunotherapy - balancing antitumor and autoimmunity. Trends Mol Med. (2019) 25:428–43. doi: 10.1016/j.molmed.2019.02.002, PMID: 30898473 PMC6488401

[40] Lu J Zhao H Yu C Kang Y Yang X . Targeting ubiquitin-specific protease 7 (USP7) in cancer: A new insight to overcome drug resistance. Front Pharmacol. (2021) 12:648491. doi: 10.3389/fphar.2021.648491, PMID: 33967786 PMC8101550

[41] Ikeda F Crosetto N Dikic I . What determines the specificity and outcomes of ubiquitin signaling? Cell. (2010) 143:677–81. doi: 10.1016/j.cell.2010.10.026, PMID: 21111228

[42] Borgeaud M Parikh K Banna GL Kim F Olivier T Le X . Unveiling the landscape of uncommon EGFR mutations in NSCLC-A systematic review. J Thorac Oncol. (2024) 19:973–83. doi: 10.1016/j.jtho.2024.03.016, PMID: 38499147

[43] Senapati J Sasaki K Issa GC Lipton JH Radich JP Jabbour E . Management of chronic myeloid leukemia in 2023 - common ground and common sense. Blood Cancer J. (2023) 13:58. doi: 10.1038/s41408-023-00823-9, PMID: 37088793 PMC10123066

[44] Roskoski R Jr . Properties of FDA-approved small molecule protein kinase inhibitors: A 2023 update. Pharmacol Res. (2023) 187:106552. doi: 10.1016/j.phrs.2022.106552, PMID: 36403719

[45] Redin E Otegui N Santos M Leon S Redrado M Serrano D . Dasatinib remodels the tumor microenvironment and sensitizes small cell lung cancer to immunotherapy. Cancer Res. (2025) 85:3910–29. doi: 10.1158/0008-5472.CAN-24-2772, PMID: 40712061

