# Supplementary figures and images for: Identification of a ubiquitin-binding domain protein, CD2AP, in predicting the prognosis and treatment of lung adenocarcinoma
Source: Front Immunol. 2025 Dec 4;16:1726531. doi: 10.3389/fimmu.2025.1726531 (PMC12711811; doi:10.3389/fimmu.2025.1726531)

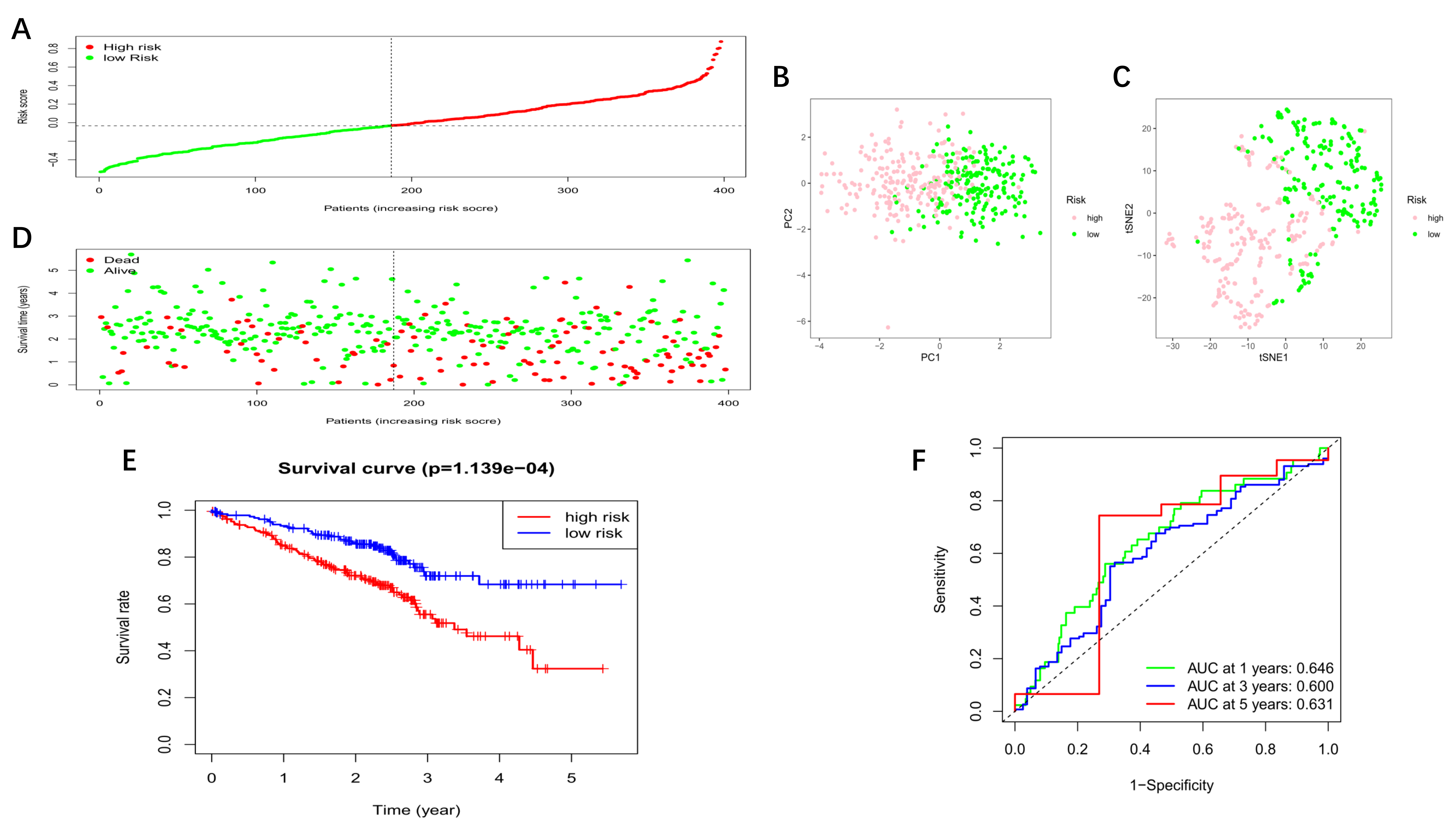

Supplement: Supplementary Figure 1 — Validation of the risk signature in an external GEO cohort. (A) The distribution of risk scores for each individual in the validation cohort. (B) The PCA plot for patients. (C) The t-SNE analysis. (D) Survival status for patients (low-risk: left of the dotted line; high-risk: right of the dotted line). (E) Kaplan–Meier curves for the OS in two risk groups. (F) Time-dependent ROC curves. [file Image1.tif]

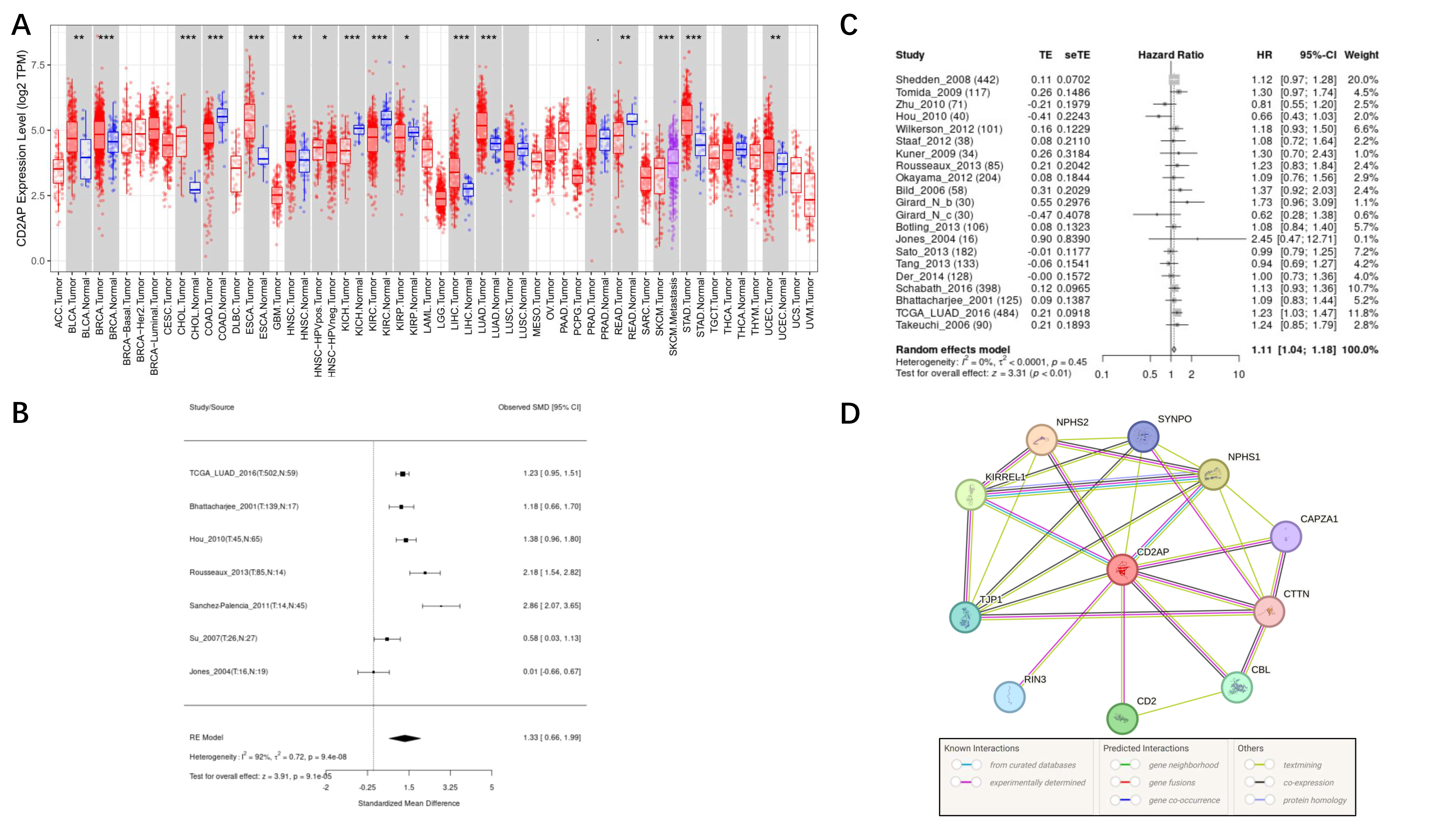

Supplement: Supplementary Figure 2 — Exploration of CD2AP functions. (A) Pan-cancer analysis comparing CD2AP mRNA levels in cancer and normal tissues. (B) Meta-analysis to compare CD2AP expression levels between normal and LUAD tissues. (C) Meta-analysis for assessing the prognostic value of CD2AP in LUAD. (D) CD2AP-centric PPI network (interaction score was set at 0.4). [file Image2.tif]

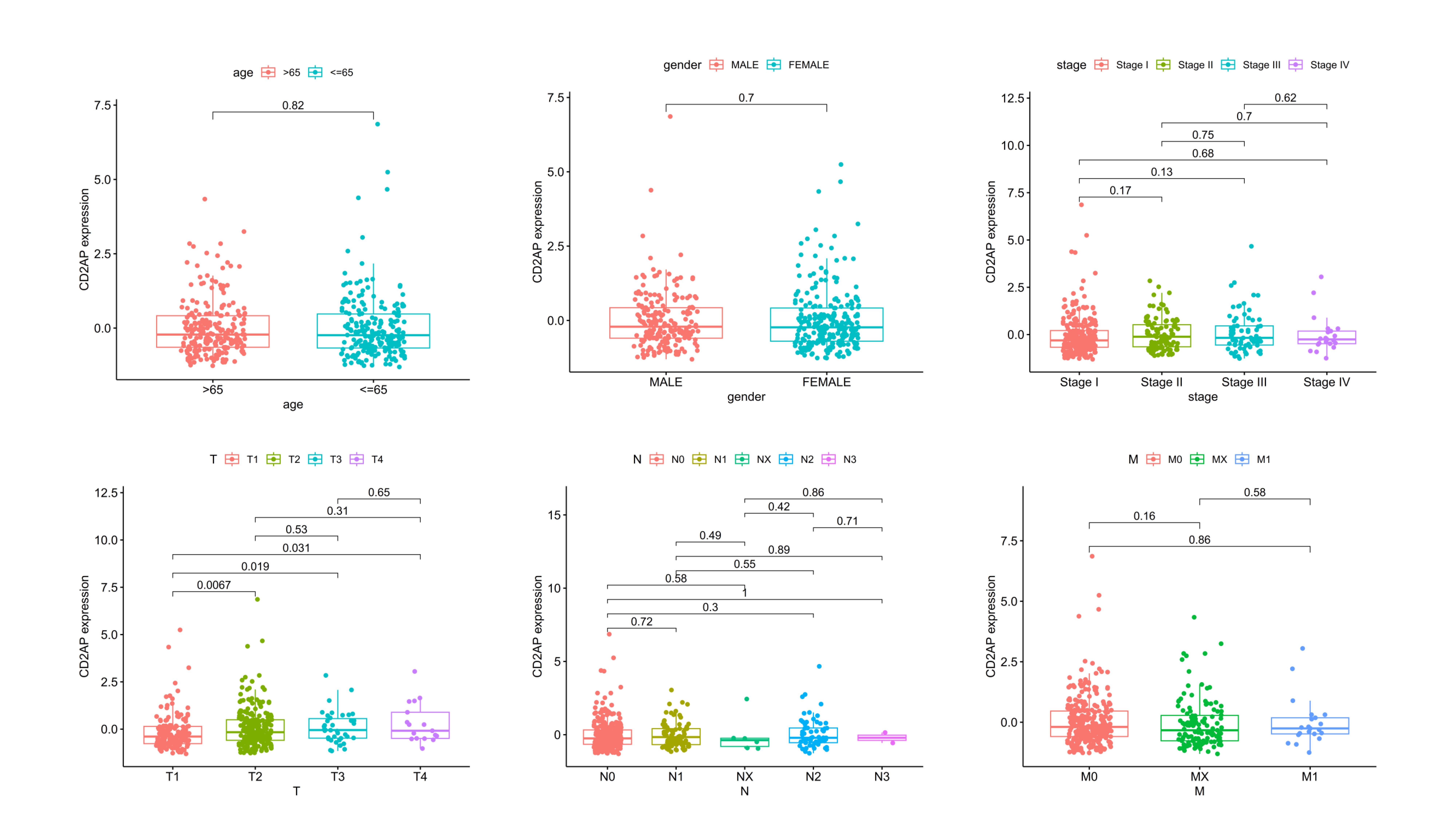

Supplement: Supplementary Figure 3 — The correlations between CD2AP expression levels and clinical features. [file Image3.tif]

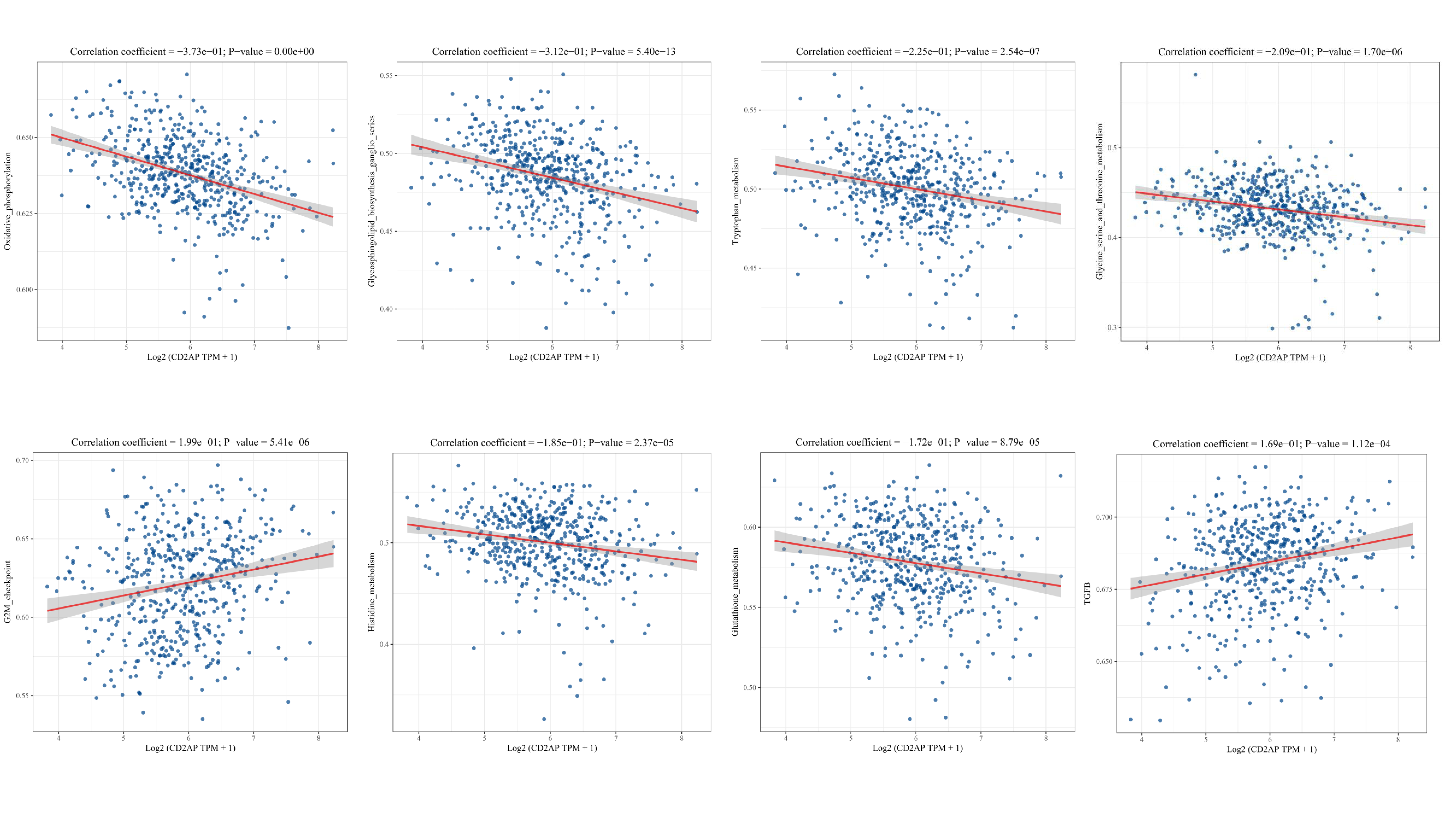

Supplement: Supplementary Figure 4 — Correlation between CD2AP expression and pathway enrichment scores derived from single-sample GSEA (ssGSEA). [file Image4.tif]

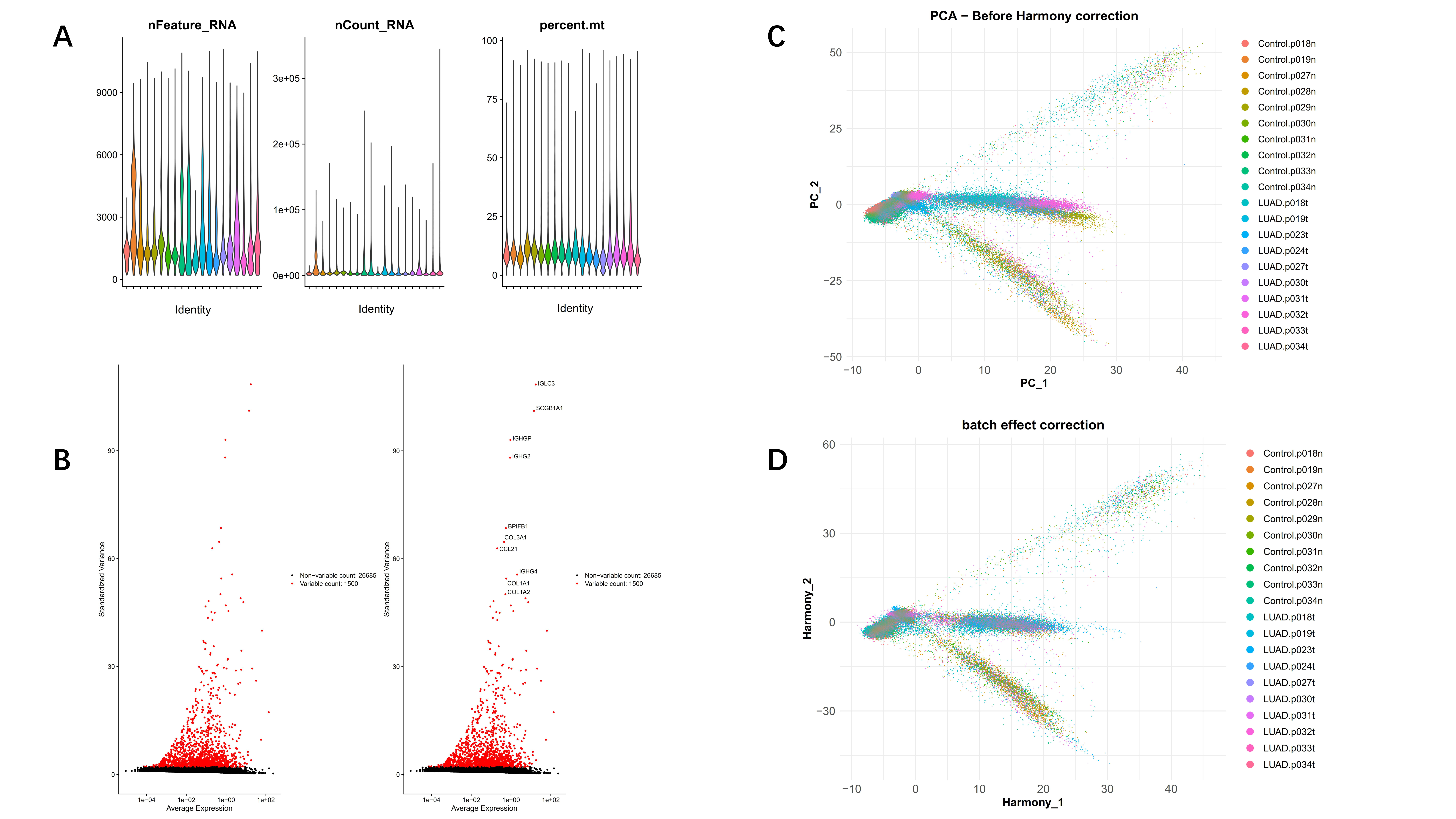

Supplement: Supplementary Figure 5 — Preprocessing and filtering of single-cell RNA sequencing data (A) the quality control metrics of sequencing data, number of detected features (nFeature_RNA), the total RNA count (nCount_RNA), the percentage of mitochondrial genes (percent.mt); (B) the percentage of haemoglobin genes; (C) PCA analysis before calibration; (D) Scatter plot after batch effect correction. [file Image5.tif]
