# Supplementary material for: Identification of a ubiquitin-binding domain protein, CD2AP, in predicting the prognosis and treatment of lung adenocarcinoma
Source: Front Immunol. 2025 Dec 4;16:1726531. doi: 10.3389/fimmu.2025.1726531 (PMC12711811; doi:10.3389/fimmu.2025.1726531)
Supplement: Supplementary Table 2 — Clinicopathological characteristics of the 13 LUAD patients in the experimental cohort. [file Table2.docx]

Table S2. Clinicopathological characteristics of the 13 LUAD patients in the experimental cohort

| **Patient ID** | **Age** | **Gender** | **Smoking History** | **TNM Stage** | **Pathological Stage** | **Sample Usage**  **(qPCR/WB/IF)** |
| --- | --- | --- | --- | --- | --- | --- |
| P1 | 71 | M | Smoker | T3N0M0 | IIB | qPCR |
| P2 | 63 | F | Non-smoker | T2aN0M0 | IB | qPCR |
| P3 | 62 | M | Smoker | T1bN1M0 | IIB | qPCR |
| P4 | 63 | F | Non-smoker | T2bN1M0 | IIA | qPCR |
| P5 | 61 | M | Smoker | T3N0M0 | IIIA | qPCR |
| P6 | 69 | F | Non-smoker | T2bN0M0 | IIB | WB |
| P7 | 81 | M | Non-smoker | T2aN2M0 | IIIA | WB |
| P8 | 48 | F | Non-Smoker | T2aN0M0 | IB | WB |
| P9 | 62 | M | Smoker | T2aN0M0 | IB | WB |
| P10 | 61 | F | Non-smoker | T2aN0M0 | IB | WB |
| P11 | 69 | M | Smoker | T2aN1M0 | IIB | IF |
| P12 | 69 | F | Non-smoker | T1cN0M0 | IA3 | IF |
| P13 | 75 | M | Non-smoker | T1cN0M0 | IA3 | IF |
